# Spinster Homolog 2 (*Spns2*) Deficiency Causes Early Onset Progressive Hearing Loss

**DOI:** 10.1371/journal.pgen.1004688

**Published:** 2014-10-30

**Authors:** Jing Chen, Neil Ingham, John Kelly, Shalini Jadeja, David Goulding, Johanna Pass, Vinit B. Mahajan, Stephen H. Tsang, Anastasia Nijnik, Ian J. Jackson, Jacqueline K. White, Andrew Forge, Daniel Jagger, Karen P. Steel

**Affiliations:** 1 Wellcome Trust Sanger Institute, Hinxton, Cambridge, United Kingdom; 2 Wolfson Centre for Age-Related Diseases, King's College London, London, United Kingdom; 3 Centre for Auditory Research, UCL Ear Institute, London, United Kingdom; 4 MRC Human Genetics Unit, MRC Institute of Genetics & Molecular Medicine, University of Edinburgh, Edinburgh, United Kingdom, and Roslin Institute, University of Edinburgh, Easter Bush, United Kingdom; 5 Omics Laboratory, University of Iowa, Iowa City, Iowa, United States of America; 6 Edward S. Harkness Eye Institute, Columbia University, New York, New York, United States of America; 7 Department of Physiology, Complex Traits Group, McGill University, Montreal, Quebec, Canada; Tel Aviv University, Israel

## Abstract

Spinster homolog 2 (Spns2) acts as a Sphingosine-1-phosphate (S1P) transporter in zebrafish and mice, regulating heart development and lymphocyte trafficking respectively. S1P is a biologically active lysophospholipid with multiple roles in signalling. The mechanism of action of Spns2 is still elusive in mammals. Here, we report that *Spns2*-deficient mice rapidly lost auditory sensitivity and endocochlear potential (EP) from 2 to 3 weeks old. We found progressive degeneration of sensory hair cells in the organ of Corti, but the earliest defect was a decline in the EP, suggesting that dysfunction of the lateral wall was the primary lesion. In the lateral wall of adult mutants, we observed structural changes of marginal cell boundaries and of strial capillaries, and reduced expression of several key proteins involved in the generation of the EP (Kcnj10, Kcnq1, Gjb2 and Gjb6), but these changes were likely to be secondary. Permeability of the boundaries of the stria vascularis and of the strial capillaries appeared normal. We also found focal retinal degeneration and anomalies of retinal capillaries together with anterior eye defects in *Spns2* mutant mice. Targeted inactivation of *Spns2* in red blood cells, platelets, or lymphatic or vascular endothelial cells did not affect hearing, but targeted ablation of *Spns2* in the cochlea using a *Sox10-Cre* allele produced a similar auditory phenotype to the original mutation, suggesting that local *Spns2* expression is critical for hearing in mammals. These findings indicate that Spns2 is required for normal maintenance of the EP and hence for normal auditory function, and support a role for S1P signalling in hearing.

## Author Summary

Progressive hearing loss is common in the human population but we know very little about the molecular mechanisms involved. Mutant mice are useful for investigating these mechanisms and have revealed a wide range of different abnormalities that can all lead to the same outcome: deafness. We report here our findings of a new mouse line with a mutation in the *Spns2* gene, affecting the release of a lipid called sphingosine-1-phosphate, which has an important role in several processes in the body. For the first time, we report that this molecular pathway is required for normal hearing through a role in generating a voltage difference that acts like a battery, allowing the sensory hair cells of the cochlea to detect sounds at extremely low levels. Without the normal function of the *Spns2* gene and release of sphingosine-1-phosphate locally in the inner ear, the voltage in the cochlea declines, leading to rapid loss of sensitivity to sound and ultimately to complete deafness. The human version of this gene, *SPNS2*, may be involved in human deafness, and understanding the underlying mechanism presents an opportunity to develop potential treatments for this form of hearing loss.

## Introduction

Spinster homolog 2 (Spns2) is a multi-pass membrane protein belonging to the Spns family. Though the functions of Spns1 and Spns3 are largely unknown, Spns2 is known to act as a sphingosine-1-phosphate (S1P) transporter, based upon previous studies in zebrafish and mouse [1]–[6]. S1P is a vital lipid. It has diverse roles, functioning as a signalling molecule regulating cell growth [7], [8], programmed cell death [9], angiogenesis [10], [11], vascular maturation [12], [13], heart development [14] and immunity [15], [16] by binding specific G-protein-coupled S1P receptors. Red blood cells and endothelial cells are important sources of circulating S1P [17]–[19]. The role of Spns2 in regulating S1P signalling is still elusive.

**Figure 1:**
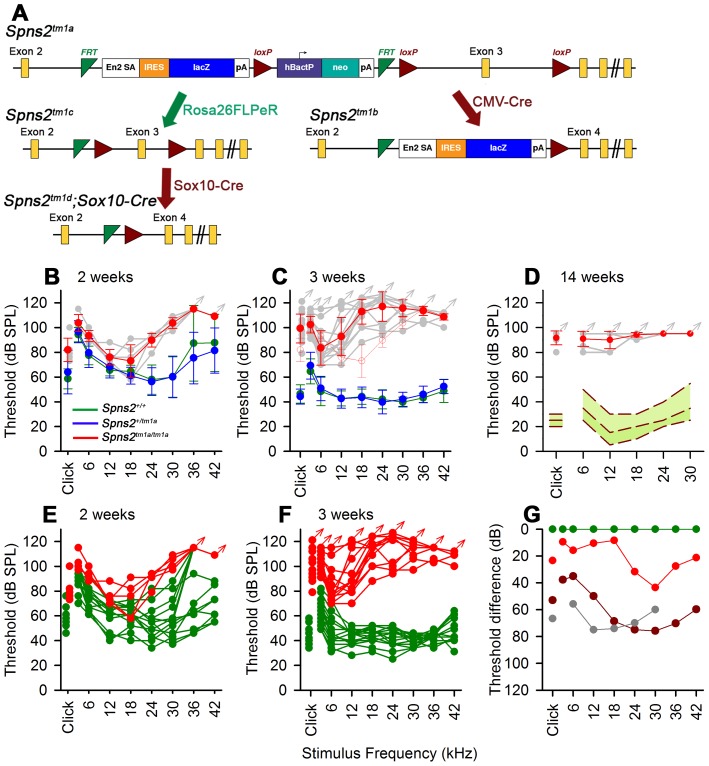
Generation and manipulation of the knockout-first allele *Spns2^tm1a^* and progressive hearing loss measured by ABR. ***A***, The promoter-driven cassette, including a splice acceptor site, an internal ribosome entry site (IRES) and a β-galactosidase reporter (lacZ), followed by a neomycin resistance marker expressed from an independent β-actin promoter, was inserted into intron2 of the *Spns2* gene. FRT sites surround the inserted cassette and *LoxP* sites flank the critical exon (exon 3 of *Spns2*; http://www.knockoutmouse.org/martsearch/project/25171). The *lacZ*-tagged *Spns2^tm1b^* allele was generated by breeding *Spns2^tm1a^*-carrying mice to mice expressing Cre recombinase driven by the CMV promoter, to delete the floxed critical exon and the neomycin-containing promoter-driven selection cassette. Flp-mediated recombination removed the main inserted cassette to convert the knockout-first allele (*Spns2^tm1a^*) to *Spns2^tm1c^*, restoring *Spns2* gene activity. Cre recombination driven by a suitable promoter such as the Sox10-Cre allele deleted the floxed exon 3 of the *Spns2^tm1c^* allele to generate a frameshift mutation (*Spns2^tm1d^*), triggering nonsense mediated decay of the transcript. ***B,C*** *Spns2^tm1a/tm1a^* mice showed progressive hearing impairment between 2 and 3 weeks of age. Raised mean ABR thresholds were detected as early as 2 weeks old in *Spns2^tm1a/tm1a^* mice (red, +/− SD), mainly from 24 kHz to 42 kHz with thresholds 30–40 dB higher than those of control mice (*Spns2^+/+^*, n = 10; *Spns2^+/tm1a^*, n = 16; *Spns2^tm1a/tm1a^*, n = 5). At 3 weeks old, hearing impairment became worse and all the frequencies were affected (*Spns2^+/+^*, n = 14; *Spns2^+/tm1a^*, n = 16; *Spns2^tm1a/tm1a^*, n = 13). Heterozygotes in blue; wildtypes in green; pale gray lines show thresholds of individual mutant mice. In ***C***, the 2 week old mean mutant thresholds are plotted in pink with open circles for comparison with 3 week old data, indicating significant progression of the hearing loss (Kruskall-Wallis One-Way Analysis of Variance on Ranks H = 102.857, 17 degrees of freedom, p<0.001). ***D***, Mean ABR thresholds of mutants (red) at 14 weeks old (n = 6, pale gray symbols represent individual mice) showed either very raised ABR thresholds or no response at all at the maximum sound level used (95 dB SPL). The green area shows the reference range for thresholds of wildtype mice of the same genetic background (n = 440), plotting the median and 2.5% to 97.5% percentiles. ***E,F***, ABR thresholds of individual *Spns2^tm1a/tm1a^* and wildtype mice. Arrows at top indicate no response at the plotted maximum sound pressure level used. Heterozygous data are comparable to those of wildtypes (green) and not shown here. ***G***, Mean thresholds of mutants at 2 weeks (red), 3 weeks (brown) and 14 weeks (gray) are shown plotted as the difference between mutant and wildtype (green) thresholds, showing an increasing difference between 2 and 3 weeks, partly due to increasing thresholds of mutants (see ***B*** and ***C***) and partly due to continuing maturation of thresholds of wildtypes.

*Spns2*-deficient mice were initially discovered to be deaf during a large-scale screen of new mouse mutants carried out by the Sanger Institute's Mouse Genetics Project (MGP). The MGP uses the KOMP/EUCOMM resource of over 15,000 genes targeted in embryonic stem (ES) cells and aims to generate new mutants and screen them for a wide range of diseases and traits to reveal the function of 160 mutant genes each year [20]. Screening of hearing using the Auditory Brainstem Response (ABR) is part of the standardised battery of primary phenotypic tests and is carried out at 14 weeks of age [20]. Mutants generated from the KOMP/EUCOMM ES cell resource normally carry LoxP and Frt sites (Fig. 1A) engineered to facilitate further genetic manipulation to generate the conditional allele and then to knock out gene expression selectively [21].

*Spns2*-deficient mice showed early onset of hearing loss that progressed rapidly to profound deafness. This was associated with declining endocochlear potential (EP), which appeared to be the primary physiological defect. At later stages we observed degeneration of sensory hair cells and decreased expression of several key genes required for normal generation of the EP in the lateral wall of the cochlea, but these appeared to be secondary effects. By producing and analysing different conditional knockouts, we established that Spns2 expression was required locally in the inner ear rather than systemically. Our study suggests a vital role for Spns2 and S1P signalling in hearing.

## Results

### *Spns2* gene targeting and mouse production

**Figure 2:**
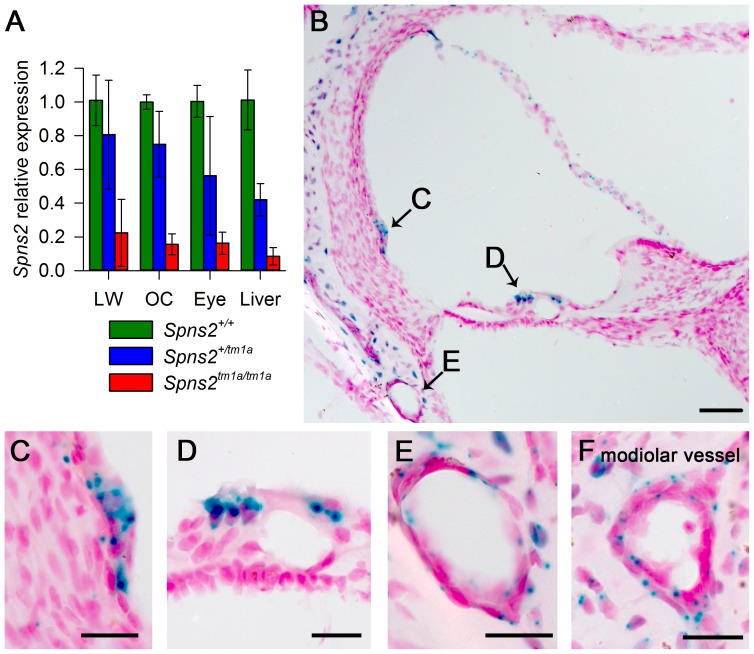
Spns2 is expressed in the cochlea. ***A***, Quantitative real time PCR showed that *Spns2* is expressed in the cochlea (OC: organ of Corti, LW: lateral wall) and other organs including the eye, and a small amount of residual transcript remained in the homozygotes (red), ranging from 8% to 22% of wildtype (green) levels. Blue represents heterozygotes. ***B***, X-gal staining showed expression of *Spns2* in the cochlea in a homozygote at P10. Labelling (blue) was detected in the spiral prominence area (***B,C***), hair cells (***B,D***), Reissner's membrane, blood vessels in lateral wall (***B,E***), modiolar vessels (***F***), and bony shell (***B***). Labelling was also seen in the central projection of the auditory nerve (not shown). Nuclei are labelled in red. Scale bar: 50 µm in C, 20 µm in D,E,F,G.

The introduction of a cassette with an additional splice acceptor site is predicted to interrupt normal transcription of the *Spns2* gene (Fig. 1A) and generate a truncated non-functional transcript encoding the first 146 out of 549 amino acids of the Spns2 protein [4]. The *Spns2^tm1a/tm1a^* mice were fertile and can survive to adulthood, but were born at sub-Mendelian ratios (15.9% homozygotes among 747 offspring of heterozygous intercrosses; χ^2^ test, *p*<0.001). Quantitative real-time PCR revealed that residual transcript of *Spns2* in cochleae, eyes and livers of the homozygous mice was substantially reduced compared to that of the heterozygous and wildtype mice (Fig. 2A). In order to completely inactivate expression of the *Spns2* gene, we produced *Spns2^tm1b/tm1b^* mice by crossing *Spns2^tm1a/tm1a^* with mice ubiquitously expressing Cre recombinase to delete exon 3 in the germline. *Spns2^tm1b/tm1b^* mice were also fertile and can survive to adulthood with a birth rate at sub-Mendelian ratios (16.9% homozygotes among 266 offspring of heterozygous intercrosses; χ^2^ test, *p* = 0.0044). In other aspects of their phenotype, *Spns2^tm1b/tm1b^* mice were broadly similar to *Spns2^tm1a/tm1a^* mice (see http://www.sanger.ac.uk/mouseportal/search?query=spns2 for a comparison of the two lines). *Spns2^tm1a/tm1a^* mice were the first to be available and were used for most experiments in this study, and may be more relevant to human disease because most disease-causing mutations reduce rather than eliminate gene activity.

### *Spns2^tm1a/tm1a^* mice have early onset progressive hearing impairment

We found that *Spns2^tm1a/tm1a^* mice had profound hearing impairment during screening at 14 weeks old by auditory brainstem response (ABR) measurement (Fig. 1D). *Spns2^tm1a/tm1a^* mice displayed no overt signs of abnormal behaviour throughout life up to 12 months old (n = 20 homozygotes, 29 control littermates) suggesting normal vestibular function. ABR measurements were recorded at younger ages to find out the time of onset of hearing loss. Hearing impairment in *Spns2^tm1a/tm1a^* mice can be detected as early as 2 weeks of age at high frequencies (Fig. 1B, E, G), and thresholds were raised further at a wide range of frequencies by 3 weeks old (Fig. 1C, F, G). The mutant thresholds recorded at P21 were significantly elevated compared with mutant thresholds at P14, indicating progressive hearing loss (Kruskall-Wallis One-Way Analysis of Variance on ranks, H = 102.857, 17 degrees of freedom, p<0.001). There was no difference in ABR thresholds between the *Spns2^+/tm1a^* and *Spns2^+/+^* mice, indicating recessive inheritance. Thresholds continue to improve in control mice from 2 to 3 weeks old as hearing function matures [22].

### *Spns2* is expressed in the inner ear

Quantitative real-time PCR showed that *Spns2* is expressed in the cochlea, both in the lateral wall and organ of Corti, and in eyes and liver in P4 wildtype mice (Fig. 2A). X-gal staining was used to indicate expression domains of *Spns2*, benefiting from the knockout first design of the allele which includes a LacZ reporter gene (Fig. 1A). At P10, X-gal labeling was detected in the spiral prominence area (Fig. 2C), hair cells (Fig. 2D), Reissner's membrane (Fig. 2B), vessels of inner ear (Fig. 2E) including the spiral modiolar vessels (Fig. 2F), proximal auditory nerve and bony shell of the cochlea (Fig. 2B), and the stria vascularis close to where the Reissner's membrane attaches. There was a similar labelling pattern at P14. The X-gal staining was also detected in the maculae and cristae in the vestibular system (Fig. S1A,B). On the basis of the expression pattern, our further investigation focused on two key components of the inner ear: the organ of Corti, where the pressure wave is transduced into action potentials, and the lateral wall, which maintains the ionic homeostasis of the cochlear endolymph.

### Progressive degeneration of organ of Corti

**Figure 3:**
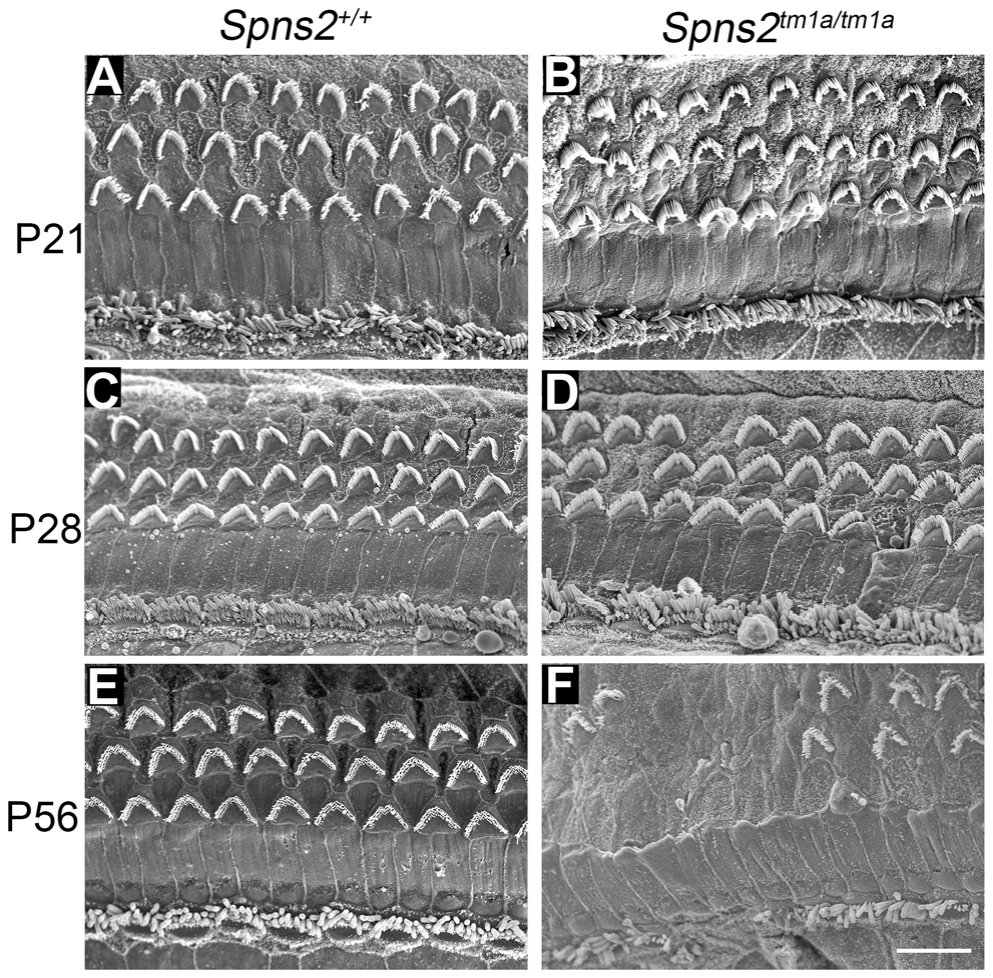
Progressive deterioration of hair cells shown by scanning electron microscopy. *Spns2^tm1a/tm1a^* mice have a normal structure of hair bundles compared with wildtype mice at P21 (***A,B***). Scattered or patchy outer hair cell (OHC) degeneration was observed in the middle turn at P28 (***C,D***). Most stereocilia of OHCs in basal and middle turn have degenerated and stereocilia of IHCs were fused or lost at P56 (***E,F***). All the images were taken from the middle turn of the cochlea, defined as 40–70% of the distance along the cochlear duct from the base. Scale bar: 10 µm.

*The Spns2^tm1a/tm1a^* mice showed a normal gross morphology of the middle ear and ossicles assessed by dissection and gross inspection, and the cleared inner ears also showed no malformation (Fig. S1C,D). We performed scanning electron microscopy (SEM) of P4, P21, P28 and P56 *Spns2^tm1a/tm1a^* mice and littermate controls. The hair cells of *Spns2^tm1a/tm1a^* mice appeared normal at P4 (Fig. S1E,F) and at P21 (Fig. 3A,B). There was scattered or patchy degeneration of stereocilia of outer hair cells in the homozygous cochleae at P28 (Fig. 3C,D). Hair cell degeneration became more apparent over time and by P56, only a few outer hair cells remained at the apex with most of them missing in other turns, and inner hair cells showed signs of degeneration such as fused stereocilia (Fig. 3E,F).

We also used transmission electron microscopy to examine the cochlear duct at P28, and observed degeneration of the basal turn organ of Corti and an apparently reduced density of dendrites in Rosenthal's canal in that turn (see later).

The increase in ABR thresholds preceded degeneration of the organ of Corti suggesting that these were secondary changes rather than the primary cause of the hearing impairment.

### Decreased endocochlear potential (EP) in *Spns2^tm1a/tm1a^* mice

**Figure 4:**
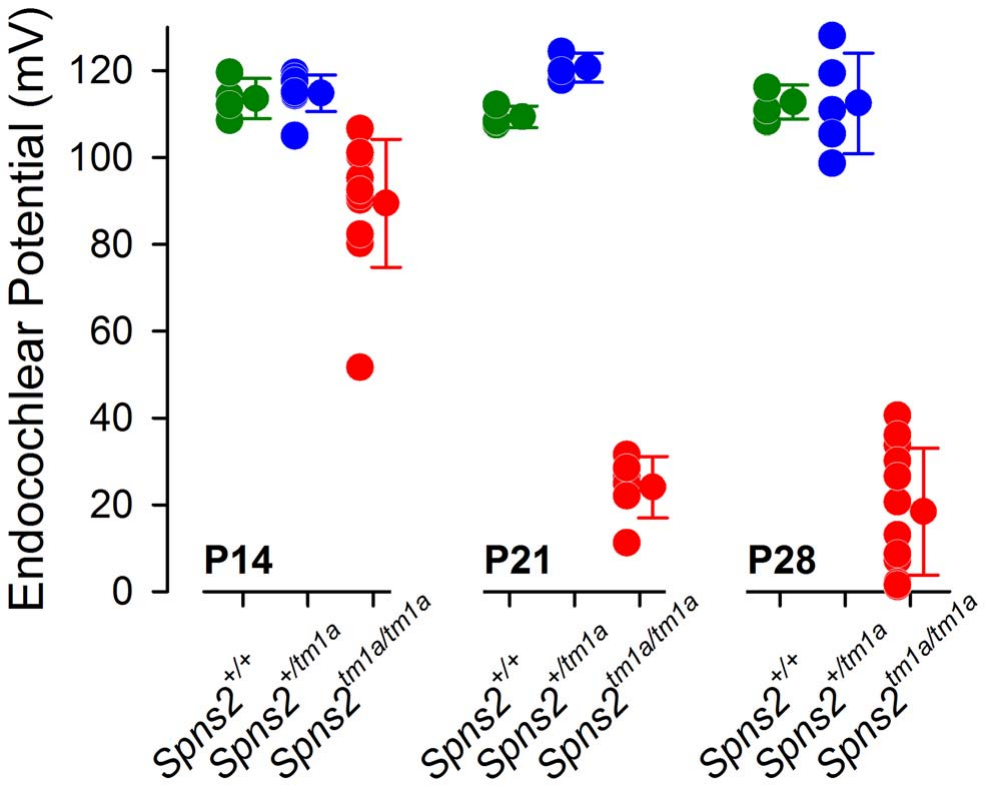
Endocochlear potential (EP) showed a progressive reduction from P14 to P21. Control mice had normal EP magnitudes of around 99–120 mV at P14 (*Spns2^+/+^*, n = 4; *Spns2^+/tm1a^*, n = 9), P21 (*Spns2^+/+^*, n = 3; *Spns2^+/tm1a^*, n = 3) and P28 (*Spns2^+/+^*, n = 4; *Spns2^+/tm1a^*, n = 5). The *Spns2^tm1a/tm1a^* mice showed a significant reduction in the EP magnitude at P14 (n = 11; p<0.001), P21 (n = 6; p = 0.002) and P28 (n = 12; p<0.001) compared to age-matched control values (Mann-Whitney Rank Sum Test; SigmaPlot v12.0). There was a large drop in the EP values in *Spns2^tm1a/tm1a^* mice between P14 and P21.

The stria vascularis is responsible for pumping K^+^ into the endolymph and generation of the endocochlear potential (EP) [23]. The EP starts to develop at around P6 in the mouse reaching adult values around P16 [24] and plays a key role in sound transduction because it provides approximately half of the electrochemical gradient that drives cations from the endolymph, a K^+^-rich extracellular fluid, into the sensory hair cells through mechanoelectrical transduction channels. The lateral wall of the cochlea is composed of the stria vascularis, spiral prominence and the spiral ligament. A defect in the function of any of these components could interfere with the generation of the EP. Therefore, we measured the EP to evaluate the function of the lateral wall. The EP values of the control mice were normal, around 99 to 120 mV, which matched their normal hearing. *Spns2^tm1a/tm1a^* mice had abnormally low EP values of 2 to 41 mV at both P21 and P28 when they were profoundly deaf (Fig. 4). However, at P14 the EP was higher, ranging from 52 to 107 mV, with some measurements within the normal range, corresponding to the partially preserved hearing at that age (Fig. 1C). The EP appeared to develop to near-normal levels and then declined very rapidly between P14 and P21. These data indicate that the cause of hearing loss in *Spns2^tm1a/tm1a^* mice is a failure to maintain the normal level of the EP after it develops, suggesting that the primary lesion is more likely to be in the lateral wall than the organ of Corti.

### Structural defects of the stria vascularis

**Figure 5:**
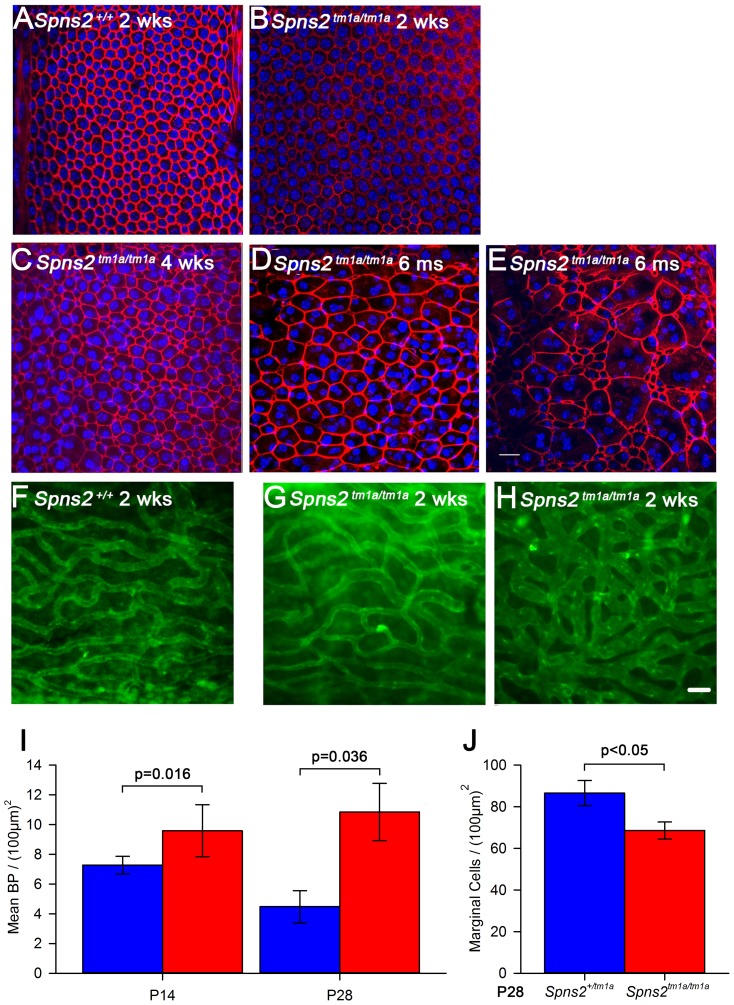
Disorganized marginal cell boundaries and abnormal strial capillaries. ***A–E***, Morphological changes of marginal cell boundaries with age. The marginal cell boundaries were visualised by phalloidin staining (red) in whole-mounts of stria vascularis. Normal morphology of the marginal cell boundaries at P14 in both wildtype (***A***) and homozygous mice (***B***), and a subtle change in homozygous mice (***C***) at P28. ***D,E*** show the further change with age in one 6-month old homozygous mouse, which displays variable changes along the length of the cochlear duct. DAPI (blue) staining visualised the nuclei. More than one nucleus can be seen within the red-labelled boundaries. Images are taken from the middle turn (40–70%) of the cochlear duct. Scale bar, 20 µm. ***F–H***, Stria vascularis showed dilated capillaries and increased branching. Strial capillaries were visualised by isolectin B4 (green). This change can be detected in patches in some mutants from P14 (***H***), but was variable (***G*** and ***H***). ***I***, Branch point counting of strial capillaries and comparison between homozygotes and controls at P14 and P28. The capillaries of homozygotes (red) at P14 (n = 5, P<0.05) and P28 (n = 5, P<0.05) had significantly increased densities of capillary branching compared with those of controls (blue). Mann-Whitney Rank Sum Test; SigmaPlot v12.0. ***J***, Density of marginal cells in surface preparations of the stria vascularis in *Spns2^tm1a/tm1a^* (red, n = 4) and *Spns2^+/tm1a^* (blue, n = 4) at P28 showed reduced density in mutants (t test, p<0.05).

In order to understand what causes the reduction in the EP, we investigated the lateral wall, where the EP is produced. Generation of a voltage difference requires efficient separation of different compartments within the cochlear duct with adequate electrical resistance. Therefore we examined the morphology of cell boundaries between adjacent marginal cells and between basal cells in whole-mount samples of the stria vascularis. Filamentous actin was stained by phalloidin to label the cell boundaries at different ages. At P14, both wildtype and homozygous mice showed a distinctive regular hexagonal pattern of the boundaries of marginal cells (Fig. 5A,B). At P28, a subtle change was seen in *Spns2^tm1a/tm1a^* mice (Fig. 5C) and it became worse with age with a patchy pattern of different layouts of cell boundaries (Fig. 5D,E). However, the boundaries were always continuous and intact in *Spns2^tm1a/tm1a^* mice without any sign of breakdown (Fig. 5C–E). We quantified the marginal cell numbers by using the labelled cell boundaries at P28. The density of marginal cells in *Spns2^tm1a/tm1a^* mice was reduced compared with controls (t test, p<0.05), associated with irregular layout of marginal cell boundaries (Fig. 5J). Noticeably, this irregularity of marginal cells was not detected in mutants at P14, a stage when hearing had started to deteriorate, but appeared at later stages. The boundaries between basal cells of the stria vascularis did not show any obvious anomalies in mutants (Fig. S2A,B).

The morphology of strial capillaries was examined as well. Some *Spns2^tm1a/tm1a^* mice showed slight dilation (2 out of 5 mice) in patches along the length of the cochlear duct, and apparently increased branching (Fig. 5H) at P14. At P28, these changes were detected in all five tested mice and were more severe than at P14 although were still patchy (Fig. S2C,D). The number of branch points per unit area in homozygotes was significantly more than that in the control mice (Mann-Whitney Rank Sum Test, p<0.05 at both P14 and P28; Fig. 5I).

**Figure 6:**
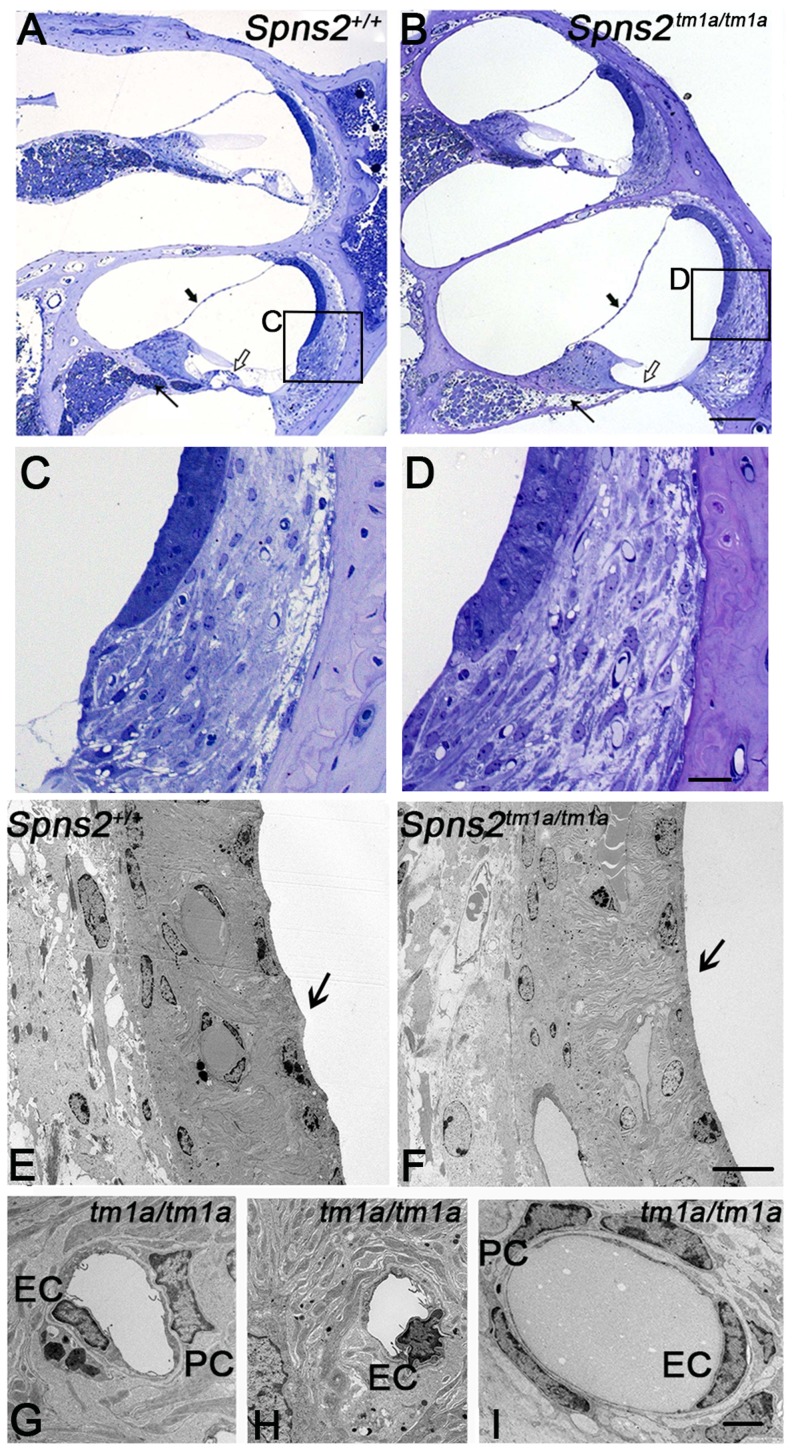
Pathological changes in stria vascularis and normal position of Reissner's membrane. ***A,B*** In semi-thin sections (P28), the position of Reissner's membrane is not changed (bold arrow). Hair cells and supporting cells of the most basal turn have degenerated (open arrow). The neural dendrites in Rosenthal's canal appeared reduced (arrow). Scale bar: 100 µm. ***C*** and ***D*** are expanded views of the areas framed in ***A*** and ***B*** and display similar morphology of fibrocytes in mutants and controls. Scale bar 20 µm. By transmission electron microscopy (P28, ***E–I***), the normal scallop-shaped (bold arrow) luminal boundary of marginal cells in control mice (***E***) was not found in the *Spns2^tm1a/tm1a^* mice (***F***). Abnormalities were seen in nuclei of endothelial cells (EC) and pericytes (PC) in strial capillaries of *Spns2^tm1a/tm1a^* mice (***G,H***), which were not seen in the capillaries of spiral ligament (***I***) and control strial capillaries (***E***). Scale bars: 10 µm in ***E,F***, 2 µm in ***G–I***.

We analysed the structure of the lateral wall of the cochlea, including the stria vascularis and spiral ligament, using semithin sections and transmission electron microscopy in P28 mice. The position of Reissner's membrane was normal in semi-thin sections of P28 cochleae (Fig. 6A,B), with no evidence of hydrops or collapse. No systematic differences in the appearance of fibrocytes of the spiral ligament were observed (Fig. 6C,D). The inner boundaries of marginal cells of the stria, facing the endolymph, have a typical scallop-shaped surface in wildtype mice with the junctions between adjacent cells raised, but this feature was not seen in the *Spns2^tm1a/tm1a^* mice and the luminal surface appeared flat (Fig. 6E,F). Nuclei of marginal and basal cells appeared more rounded in *Spns2^tm1a/tm1a^* mice than in wildtypes (Fig. 6E,F). There was also a marked difference in the appearance of endothelial cells and pericytes [25], [26] of strial capillaries, with the nuclei of mutant cells appearing more darkly-stained (Fig. 6G,H). However, this abnormality appeared to be limited to capillaries of the stria vascularis only, and was not seen in the spiral ligament capillaries (Fig. 6I), suggesting a specific effect of Spns2 deficiency on the capillaries of the stria vascularis.

Intermediate cells of the stria vascularis are derived from melanocytes and tend to accumulate pigment during ageing or under stress such as in mice with Pendrin deficiency [27], [28]. The pigmented cells may derive from migratory melanocytes that adopt macrophage-like features during development [27], [29] or may derive from macrophage invasion [28]. At 4 weeks of age, we did not observe any obvious difference in strial pigmentation between *Spns2* mutants and control littermates, but by 7 months old the mutant strias appeared more strongly pigmented than controls (Fig. S2E,F). This timecourse, after the onset of raised thresholds, suggests the accumulation of pigment is likely to be a secondary effect, not a cause of cochlear dysfunction.

### Normal strial integrity and normal permeability of strial capillaries to BSA-FITC

**Figure 7:**
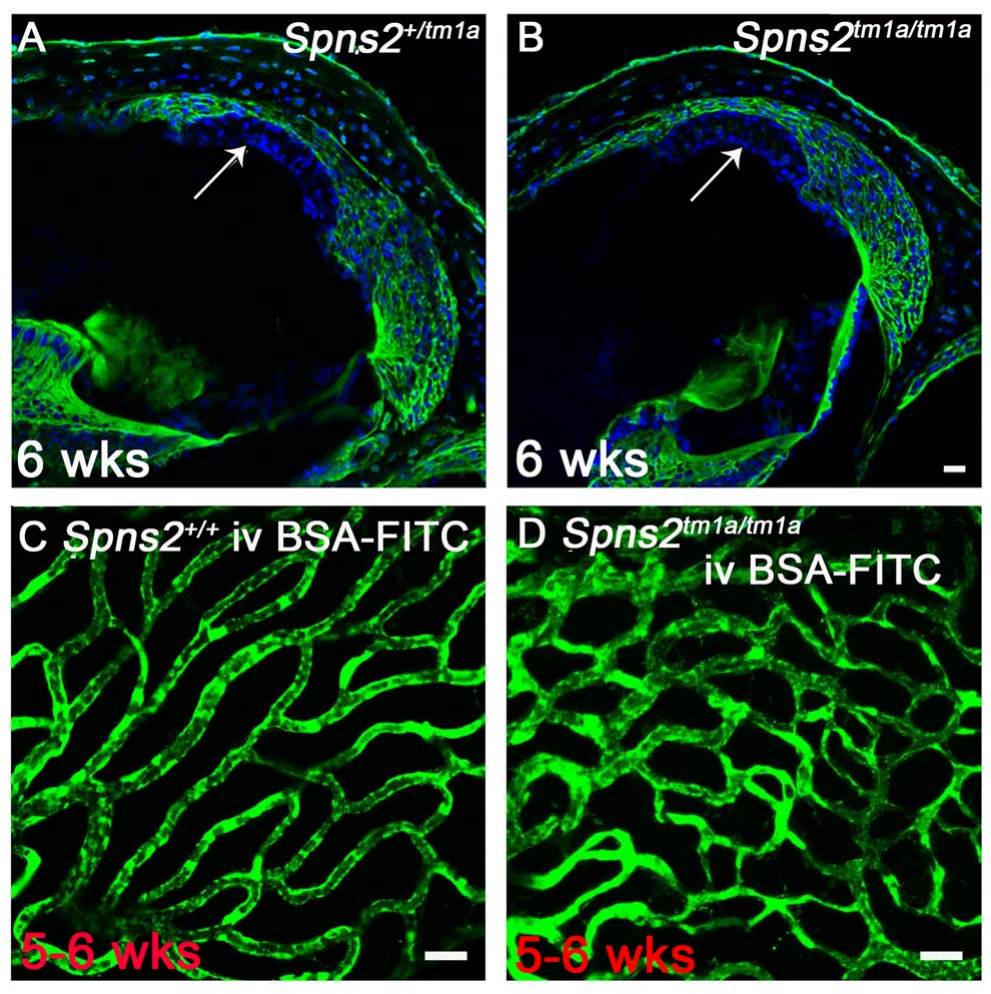
Normal strial integrity and normal permeability of strial capillaries to BSA-FITC. ***A,B***, Endolymphatic and perilymphatic compartments were perfused by Sulfo-NHS-LC-Biotin. Biotin was detected by FITC-conjugated streptavidin (green) in frozen sections of 6 week old mice. No sign of biotin entry into the stria vascularis compartment was found as shown by the arrow indicated that the tight junctions of marginal and basal cells are sealed in *Spns2^tm1a/tm1a^* mice (***B***) compared with the control mice (***A***). ***C,D***, Stria vascularis capillaries of young adult wildtype (***C***) and *Spns2^tm1a/tm1a^* (***D***) mice following BSA-FITC injection into the tail vein, showing no evidence of leakage of the tracer (green) out of the capillaries. The increased branching of capillaries in these *Spns2^tm1a/tm1a^* mice is also visible. Scale bars, 20 µm in ***A–D***.

In view of the abnormal morphology of marginal cell boundaries, we asked whether the diffusion barrier of stria vascularis, for example between adjacent marginal cells, was affected because normal morphology of boundaries at P14 does not necessarily mean normal function. We used biotin as a tracer injected into the endolymphatic and perilymphatic compartments of 6 week old mice to test the barrier permeability of the stria vascularis. There was no evidence of biotin entry into the stria vascularis of *Spns2^tm1a/tm1a^* or control mice indicating a normal diffusion barrier of stria vascularis (Fig. 7A,B). As we observed dilated strial capillaries with abnormal endothelial cells and pericytes, we tested their permeability by injecting BSA-FITC into the tail vein. There were no signs of leakage of the tracer to the tissues surrounding the strial capillaries in *Spns2^tm1a/tm1a^* mice suggesting that they have normal permeability to BSA-FITC (Fig. 7C,D).

### Decreased expression of Kcnj10, Kcnq1, Gjb2 and Gjb6 in lateral wall

**Figure 8:**
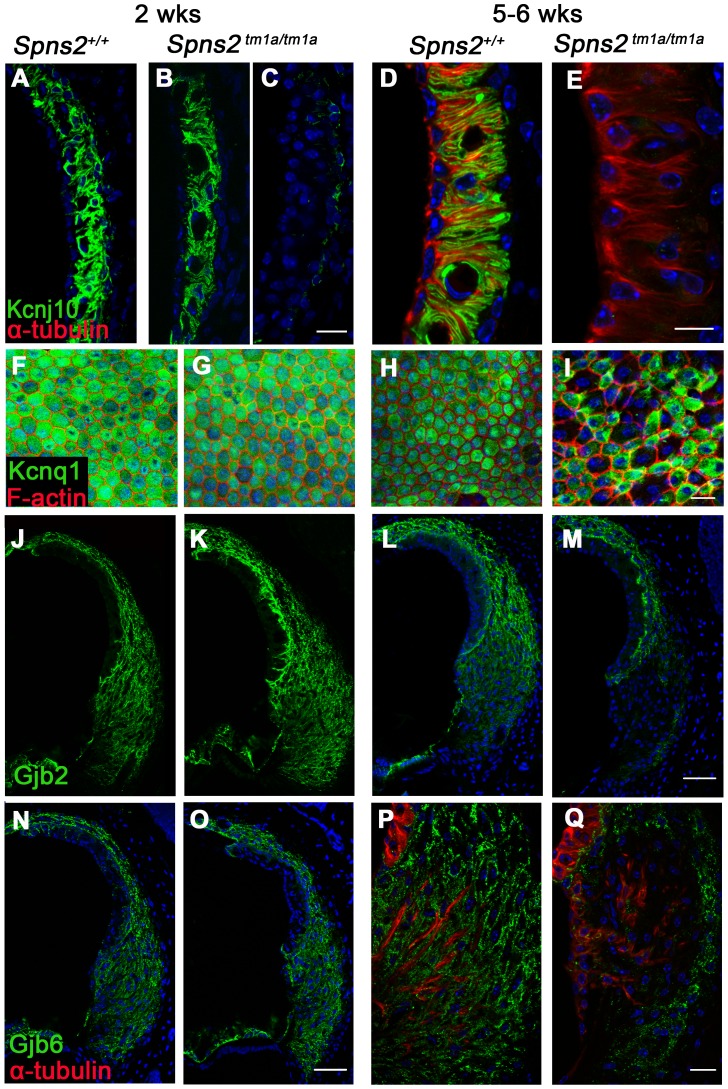
Progressive decrease in expression of Kcnj10, Kcnq1, Gjb2 and Gjb6 in *Spns2^tm1a/tm1a^* mice. ***A–E***: At P14, Kcnj10 expression (green) of homozygotes was comparable to that of wildtype in apical turns, while in basal turns, some appeared normal (***B***) as wildtype (***A***), but some appeared largely reduced (***C***). At 5–6 weeks old, Kcnj10 labelling was absent in homozygotes (***E***). Acetylated α-tubulin (red) was used to label strial marginal cells in ***D,E***. ***F–I***: Whole mount preparations of the stria. Kcnq1 labelling (green) was detected at P14 in both homozygotes (***G***) and wild types (***F***), but it was absent from those marginal cells with enlarged cell boundaries in *Spns2^tm1a/tm1a^* mice at 5–6 wks (***I***). Phalloidin (red) labelled filamentous actin to reveal the boundaries of marginal cells. ***J–Q***: Gjb2 and Gjb6 were present in the fibrocytes of the spiral ligament in both wild type and *Spns2^tm1a/tm1a^* mice at P14 (***J,K,N,O***). At 5–6 wks, expression was absent in the area behind the spiral prominence corresponding to the type II fibrocytes in homozygotes (***M*** and ***Q***) compared with wildtypes (***L*** and ***P***) of the same age. Root cells were labelled by acetylated α-tubulin (red) in ***P,Q***. DAPI (blue) labelled the nuclei. Scale bar, 10 µm in ***D,E***. 20 µm in ***A–C***, ***F–I,P,Q***. 50 µm in ***J–O***.

To further investigate the reasons underlying the reduced EP and deafness in the *Spns2 ^tm1a/tm1a^* mice, we analysed expression of some key proteins involved in normal EP formation and maintenance by immunofluorescence labelling, including Kir4.1 (*Kcnj10*), Kv7.1 (*Kcnq1*), Cx26 (*Gjb2*), Cx30 (*Gjb6*), Na^+^, K^+^-ATPase (*Atp1a1)*, NKCC1 (*Slc12a2*) and ZO-1 (*Tjp1*). In homozygotes aged P14 (Fig. 8F,G,J,K,N,O), the expression of these proteins appeared normal, apart from the expression of Kcnj10, which appeared normal in most mutants (5/8), with the remaining three mice showing reduced labelling in the basal turn only (Fig. 8A–C). At 5–6 weeks old, we observed similar expression of Na^+^/K^+^-ATPase, NKCC1 and ZO-1 in *Spns2 ^tm1a/tm1a^* mice compared with control mice (Fig. S3A–F). However, the other four proteins showed reduced expression at this age and the expression of Kcnj10 was largely absent in the stria vascularis (Fig. 8D,E). In contrast, there was similar labelling intensity of Kcnj10 in the satellite cells of the spiral ganglion in mutants and control mice (Fig. S3G,H). The expression of Kcnq1 appeared to be evenly distributed on the luminal surface of marginal cells in both wildtype and homozygotes at P14 (Fig. 8F, G), but at 5–6 weeks, labelling of Kcnq1 in the homozygotes was absent in some of the marginal cells that had enlarged boundaries (Fig. 8H,I). There was extensive expression of Gjb2 and Gjb6 in fibrocytes type I, II, and V of the spiral ligament in the wildtype (Fig. 8J,L,N,P) and homozygotes (Fig. 8K,O), but expression appeared greatly reduced behind the spiral prominence area corresponding to the fibrocyte type II region [30] in the *Spns2^tm1a/tm1a^* mice (Fig. 8M,Q) aged 5–6 weeks old. As EP started to reduce at P14, while expression of Kcnj10 (in the majority of observed mice), Kcnq1, Gjb2 and Gjb6 appeared normal at this age, these findings suggest that the reduction in expression of these key proteins in adults is secondary to a primary dysfunction of EP generation.

### Conditional knock-out of *Spns2* suggests a local function

**Figure 9:**
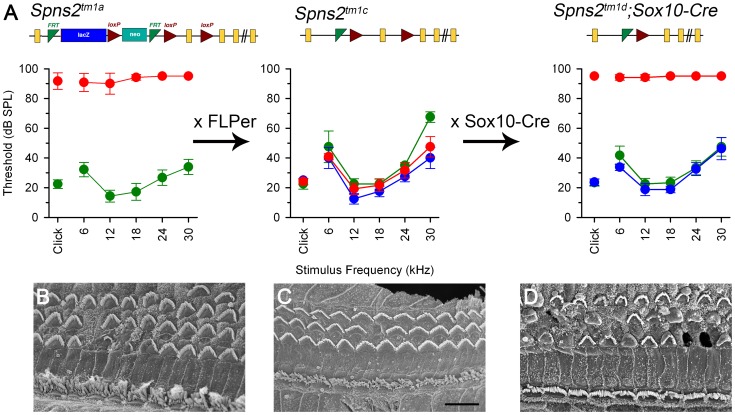
ABR thresholds and SEM assessment suggest a local function of Spns2 in the inner ear. ABR thresholds (means +/−SD) are shown for homozygotes (red), heterozygotes (blue) and wildtypes (green), aged 7–14 weeks. Mice homozygous for the *Spns2^tm1a^* allele displayed elevated ABR thresholds and degeneration of hair cells (***A*** *left*, *4 wks and* ***B***). By crossing with mice expressing Flp recombinase to excise the inserted cassette, *Spns2^tm1c/tm1c^* mice were produced, which had normal ABR thresholds and normal hair cell morphology (***A*** *middle*, *8 wks and* ***C***). Then *Spns2^tm1c/tm1c^* were crossed with *Sox10-Cre* mice to produce *Spns2^tm1d/tm1d^*;Sox10-Cre mice which showed no response up to 95 dB SPL and hair cell degeneration with bulges and holes in the reticular lamina (***A*** *right*, *4 wks and* ***D***). SEM images are taken from the middle turn (40–70%) of the cochlea. Scale bar: 10 µm in ***B,C,D***.

We generated the conditional allele of *Spns2* (*Spns2^tm1c^*) by crossing the *Spns2^tm1a^* allele to a line expressing Flp recombinase to excise the inserted cassette (Fig. 1A). The *Spns2^tm1c^* ^/*tm1c*^ mice have the same *Spns2* allele as the wildtype except that exon 3 is flanked by two *loxP* sites. *Spns2^tm1c^* ^/*tm1c*^ mice showed normal ABR thresholds and normal morphology of hair cells (Fig. 9A, C). These observations confirmed that the inner ear defects we found in the *Spns2^tm1a/tm1a^* mice were due to the insertion of the cassette and its disruption of *Spns2* gene function.

**Figure 10:**
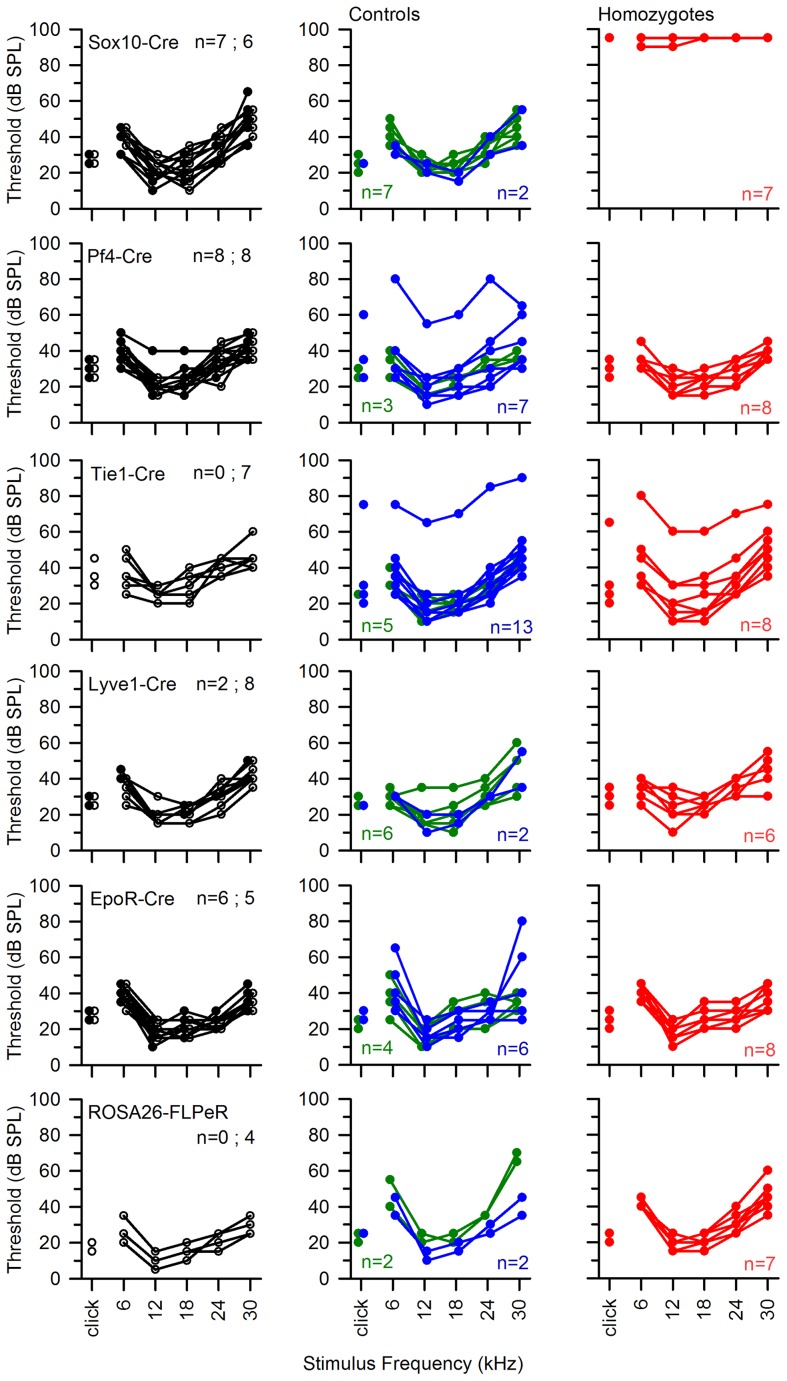
ABR thresholds of mice with *Spns2* conditionally inactivated in different tissues. ABR thresholds of individual mice are shown. All Cre driver lines showed normal thresholds (left column, in black. n = number of wildtype; number of mice carrying Cre). Most control littermates had normal responses (middle column: heterozygotes in blue; wildtypes in green). Homozygous *Spns2^tm1d^* mutants (red) carrying the relevant Cre alleles are shown in the right column. *Spns2^tm1d^* homozygotes carrying the Sox10-Cre allele had raised thresholds (top right), but the other four Cre lines had normal thresholds. The bottom row shows equivalent threshold data for the *Spns2^tm1c^* allele and the Flp recombinase line used to generate this allele, again showing normal thresholds. There were three exceptions of individuals with raised thresholds (one heterozygote each in Pf4-Cre cross and Tie1-Cre cross, one homozygote in Tie1-Cre cross), which we believe probably carry an independent mutation causing the impairment (subject to ongoing positional cloning study).

**Figure 11:**
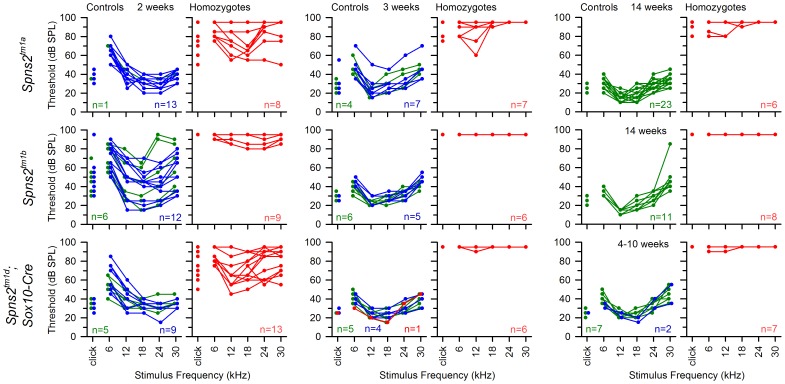
The hearing loss of *Spns2^tm1a/tm1a^*, *Spns2^tm1b/tm1b^* and *Spns2^tm1d/tm1d^*; *Sox10-Cre* mice showed a similar pattern of progression. ABR thresholds of individual homozygotes (red), heterozygotes (blue) and wildtypes (green) are shown at 2, 3 and 14 or 4–10 weeks old. The control mice had immature thresholds at 2 weeks old and continued to mature to normal hearing levels at 3 weeks old. *Spns2^tm1a/tm1a^* and *Spns2^tm1d/tm1d^*; *Sox10-Cre* mice displayed progressive hearing loss from 2 to 3 weeks old. The red line in the middle bottom panel represents a control mouse homozygous for *Spns2^tm1c^* but without carrying *Sox10-Cre*, and thus had no conditional knockout of *Spns2* in the inner ear and normal hearing.

We then asked whether the hearing defects of *Spns2^tm1a/tm1a^* mice are caused by localised deficiency of Spns2 in the inner ear or are mediated systemically. S1P is known to be released from several other tissues that could affect cochlear function, including various blood cell types and endothelial cells [1], [2]. We generated conditional knockout mice carrying the *Spns2^tm1d^* allele in specific tissues by crossing mice carrying the *Spns2^tm1c^* allele with mice carrying Cre recombinase under the control of five different promoters: Tie1, Pf4, Lyve1, EpoR and Sox10, driving expression of Cre recombinase in blood vessel endothelial cells, platelets, lymphatic endothelial cells, red blood cells, and the inner ear with surrounding neural crest-derived mesenchyme respectively. *Sox10-Cre* transgenic mice have been successfully used to express Cre recombinase in the developing inner ear previously [31]. Homozygous *Spns2^tm1d^* mutants carrying the Tie1, Pf4, Lyve1 and EpoR Cre alleles all had normal ABR thresholds in young adults (Fig. 10). In contrast, no ABR response was detected in the young adult *Spns2^tm1d/tm1d^*;*Sox10-Cre* mice (Fig. 10). *Spns2^tm1d/tm1d^*; *Sox10-Cre* mice showed a similar pattern of progression of raised thresholds between 2 and 3 weeks old as observed in *Spns2^tm1a/tm1a^* mice (Fig. 11). *Spns2^tm1d/tm1d^*; *Sox10-Cre* mice also showed similar inner ear pathological changes as found in *Spns2^tm1a/tm1a^* mice, such as degeneration of hair cells (Fig. 9B, D) and irregular arrangement of marginal cell boundaries. Therefore, we propose that *Spns2* plays an important role in mammalian hearing through its localised function in the inner ear.

The *Spns2^tm1b/tm1b^* mutants, with deletion of exon 3 of *Spns2*, showed a more severe increase in thresholds than the *Spns2^tm1a/tm1a^* and *Spns2^tm1d/tm1d^*;*Sox10-Cre* mice from 2 weeks old, the earliest stage studied, with a lack of detectable response at most frequencies in most mice up to the maximum stimulus intensity used, 95 dB SPL (Fig. 11, middle row).

### Eye defects

**Figure 12:**
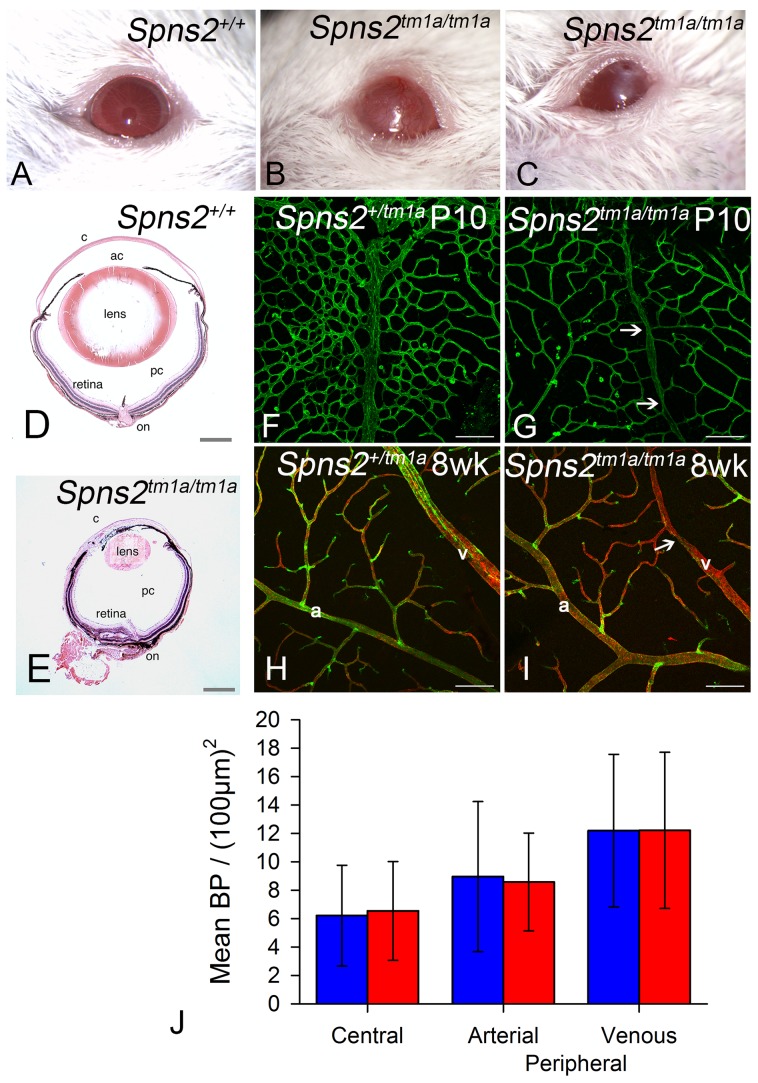
Eye defects in *Spns2^tm1a/tm1a^* mice. ***A–C***, Phenotype screening was performed on 15 week old *Spns2^tm1a/tm1a^* mice and identified buphthalmos, corneal opacity with vascularization and possible ulceration (***B***) and corneal opacity with vascularisation and polyp, thick discharge, elongated pupil which did not fully dilate with tropicamide (***C***). Albino mice were selected to achieve clearer demonstration, and a wildtype is shown in (***A***). ***D***, Pupil-optic nerve section of a 15 week old wildtype eye. ***E***, The *Spns2^tm1a/tm1a^* mice showed a grossly abnormal eye with corneal opacity, vascularization, collapsed anterior chamber, small cataractous lens, and focal retinal degeneration. c = cornea, ac = anterior chamber, pc = posterior chamber, on = optic nerve. Scale bar = 450 µm. ***F,G***,***H,I*** Analysis of retinal vasculature was performed on retinal wholemounts from P10 pups (***F,G***) and 8 week old adult mice(***H,I***). Retinal vasculature was stained with Isolectin B4 (green) to visualise the endothelium and Proteoglycan NG2 (red) to visualise pericytes. Whereas arteries (a) appeared morphologically normal, veins (v) appeared thinner in *Spns2^tm1a/tm1a^* (***I***) than in *Spns2^+/tm1a^* (***H***) and had an irregular caliber with regions of narrowing (arrows). Although the retina has three capillary plexi only the primary plexus is shown at both P10 and 8 weeks as this is the first plexus to form and mature. Scalebars: 50 µm. ***J***, Branch point analysis was performed on P10 retinal vasculature (***F,G***) to determine whether retinal vasculature showed any developmental abnormalities in vascular patterning. No significant difference was detected between *Spns2^tm1a/tm1a^* (red) and *Spns2^+/tm1a^* (blue) mice in the central retina (mature vessels) or the periphery, where the vessels are still developing at P10, in either the arteries or veins (Mann-Whitney U test for central branch points, p = 0.69; t-test for peripheral branch points, arterial p = 0.899, venous p = 0.996).

*Spns2^tm1a/tm1a^* mice had other defects detected by the MGP phenotyping pipeline such as low white blood cell count and increased bone mineral density [4], [20], [32]. However, the eye defects were of particular interest because of the relatively common association of retinal defects with deafness, as in Usher syndrome for example, and our finding of *Spns2* expression in the eye (Fig. 2A). We assessed the retina for features corresponding to those found in the organ of Corti, and found focal degeneration of the retina (Fig. 12D,E). As focal degeneration can be associated with a *Crb1^rd8^* mutant allele found in some C57BL/6 lines [33], we sequenced this gene and confirmed that the *Spns2* retinal phenotype was independent of the *rd8* mutation. As the *Spns2^tm1a/tm1a^* stria vascularis capillaries showed abnormal morphology, we examined the retinal vasculature in whole mount preparations. Retinal vein morphology also appeared abnormal with some veins appearing thinner in mutants than in controls as well as veins of irregular caliber (Fig. 12F–I). This retinal vascular phenotype was first evident by P10 when the retinal vasculature was still undergoing development, and persisted into adulthood. We therefore undertook branchpoint analysis to quantify any differences between *Spns2^tm1a/tm1a^* and *Spns2^+/tm1a^* mice. This showed no difference between genotypes at P10 (Fig. 12J). We also analysed the pericyte coverage of the retinal vessels, as a reduction in pericyte coverage is associated with increased vascular permeability. We found no significant difference in pericyte coverage in peripheral vessels, and a small but significantly reduced coverage in central vessels (Fig. S4A). The vitreous and optic nerve appeared normal in the mutants (Fig. 12D,E).

Other eye defects included open eyelids at birth (Fig. S4B) resulting in corneal opacity, vascularization and ulceration (Fig. 12A,B,C). These corneal defects made retinal assessment by ophthalmoscopy impossible *in vivo*. Histological examination showed corresponding gross morphological defects. Eyes were smaller, the cornea was thickened with vascularisation, the anterior chamber was collapsed, and the lens was small and cataractous (Fig. 12D,E). We observed the anterior eye defects in the *Spns2^tm1a/tm1a^* and *Spns2^tm1b/tm1b^* mice, but these defects were not seen in *Spns2^tm1d/tm1d^*;*Sox10-Cre* mice or any of the other four conditional lines.

## Discussion

Here, we report that *Spns2*-deficient (*Spns2^tm1a/tm1a^*) mice have profound hearing loss and propose an underlying mechanism: a rapid decline in EP paralleling loss of auditory sensitivity and preceding degeneration of hair cells, suggesting that the primary lesion is in the cochlear lateral wall, the site of EP production and maintenance [23], [34], [35]. Reduced EP has been associated with ion transport defects [36]–[39]; defects of tight junctions [40] or gap junctions [41]–[43]; absence of melanocytes [24]; microvascular disease [44], [45]; abnormal spiral ligament development [35]; sphingomyelin metabolic disturbance [46], or the lateral wall can simply be a target in systemic diseases [47]. This suggests complexity underlying the strial/metabolic category of hearing loss described in humans [48]. EP is essential for hair cell function [49]. Degeneration of hair cells secondary to reduced EP has been reported in other mouse mutants [50], [51] and normal EP seems to be important for survival of the hair cells. However, as *Spns2* is expressed in hair cells as well as in the lateral wall, we cannot exclude the possibility that disruption of Spns2 function in the organ of Corti also contributed to raised ABR thresholds and hair cell degeneration. Analysis of mice with conditional knockout of *Spns2* in hair cells and other cochlear cell types will be useful in dissecting the role of *Spns2* further.

Spns2 acts as a transporter of S1P [1]–[6]. S1P may modulate vascular tone [52] and has been shown to regulate the inner ear spiral modiolar artery tone *in vitro* [53], [54]. S1P-induced vasoconstriction is thought to be important to protect strial capillary beds from high pressure [54]. We found that *Spns2* was expressed in blood vessels of the inner ear including spiral modiolar vessels. Any reduction in local S1P level due to Spns2 dysfunction may weaken vasoconstriction and explain the dilation of strial capillaries in *Spns2^tm1a/tm1a^* mice. The relationship between capillary size and EP value is not unidirectional; both smaller and larger strial capillaries have been reported in different mouse mutants with low EP [55], [56]. S1P signalling also can affect vascular permeability [57]–[59]. However, we did not see increased permeability of strial capillaries using BSA as a tracer. Recently, Mendoza and colleagues found little difference in lung vascular permeability between *Spns2*-deficient and control animals [2], similar to our finding in strial capillaries of *Spns2^tm1a/tm1a^* mice. BSA is a medium molecular mass tracer (66.4 kDa), so tracers of different sizes and properties such as Evans blue and cadaverine [29] may be useful for further investigation of the strial capillary barrier.

We found decreased expression of several proteins critical for normal EP production at 5–6 weeks of age in *Spns2*-deficient mice. However, the expression of most of these proteins appeared normal at the time when the EP has already started to decline at P14, suggesting that these are likely to be secondary effects. The morphological changes of marginal cell boundaries and reduction in marginal cell density together with a lack of expression of Kcnq1 in affected cells are also likely to be secondary changes because these features were normal when hearing started to deteriorate at P14 and they did not affect strial permeability. Loss of Kcnq1 expression in marginal cells with expanded luminal surfaces may be a common consequence of strial dysfunction because it has been reported in several different mutants with reduced EP [28], [46]. It has been suggested that these common changes in mutant marginal cells may occur because these cells are relatively sensitive to metabolic stress [60], [61]. The variable decrease in Kcnj10 labelling in the basal turn and slightly dilated strial capillaries in a few mutants at P14 were the earliest abnormalities we have seen, and may correlate with the variable reduction in EP values at the same age. Disrupted expression of Kcnj10 is another common consequence of strial dysfunction [37], [47], [62]. The causal direction between reduced EP and decreased Kcnj10 expression in *Spns2*-deficient mice needs further investigation.

The most robust labeling for *Spns2* expression was consistently in the hair cells and spiral prominence. The function of the spiral prominence is unclear. Two types of voltage-dependent K^+^ currents are expressed in spiral prominence epithelial cells, which may play a role in the homeostasis of inner ear fluids [63]. Another gene expressed strongly in the spiral prominence is pendrin (*Pds*), and the *Pds* mutant mouse also shows reduced EP [64], loss of expression of Kcnj10 [37] and Kcnq1 [28], and increased accumulation of strial pigmentation compared with controls [28]. However, the *Pds* mutant shows a severe early developmental defect of the inner ear with extensive hydrops [65], which we do not find in *Spns2* mutants. This indicates that *Spns2*-deficient and *Pds*-deficient mice may have different mechanisms underlying the reduced EP and hearing impairment. Dysfunction of the spiral prominence in *Spns2*-deficient mice may be the main trigger of reduction of the EP and a series of pathological changes in inner ears.

One later change we saw in the lateral wall was a localised decrease of Gjb2 and Gjb6 expression in the type II fibrocytes of the spiral ligament located adjacent to the spiral prominence. Type II fibrocytes are important for K^+^ recycling and are considered to mediate K^+^ translocation between the epithelial cell network of the organ of Corti and the fibrocyte network of the lateral wall, and to facilitate ion flow directed towards the stria vascularis [66]. Intriguingly, this reduction in expression was seen for Gjb2 and Gjb6 only, and no reduction in labelling was found of Atp1a1 and Slc12a2, two other proteins strongly expressed in type II fibrocytes [67], [68]. This may indicate a selective impact of Spns2 deficiency on Gjb2 and Gjb6 expression in nearby cells, or alternatively these two genes may be more sensitive to changes in homeostasis in the cochlear duct than Atp1a1 and Slc12a2.

S1P is a bioactive lipid and acts as a second messenger intracellularly and as a ligand for cell surface G protein-coupled receptors extracellularly [69]. Five different S1P receptors participate in cellular responses based on the cell type and available downstream effectors [70]. S1P signalling has been implicated in maintenance of hair cells via activation of S1P receptor 2 (S1PR2) [71]. *S1pr2*-null mice are deaf and share some pathological changes with *Spns2*-deficient mice, such as disorganized cell boundaries of marginal cells, dilated capillaries in the stria vascularis, and degeneration of the organ of Corti [54], [71], [72]. Unlike *S1pr2*-null or *S1pr2*/*S1pr3* double null mice, no overt vestibular defects were found in *Spns2*-deficient mice. Thus, we propose that the Spns2-S1P-S1PR2 signalling axis is important for normal hearing function. A similar Spns2-S1P-S1PR2 signalling axis may exist in bones as both *S1pr2*-deficient [73] and *Spns2-*deficient [32] mice have strong but brittle bones with high bone mineral density. In contrast, the Spns2-S1P-S1PR1 signalling axis is more important for lymphocyte trafficking [1]–[5].

Systemic disruption of *Spns2* function in blood vessel or lymphatic endothelial cells, platelets or red blood cells did not affect hearing, suggesting that systemic loss of Spns2 activity in these tissues does not mediate the hearing loss we see in the *Spns2^tm1a^* mutants. However, when we deleted *Spns2* locally in the inner ear using the Sox10-Cre recombinase, the resulting mutants were deaf. *Sox10* is expressed throughout the otic epithelium from an early stage of development as well as in cranial neural crest-derived cells, so can effectively drive deletion of exon 3 of the *Spns2^tm1c^* allele in the entire inner ear [74], [75]. These findings indicate that hearing loss in *Spns2^tm1a/tm1a^* mice is due to local loss of Spns2 function in the inner ear.

Defects of the anterior eye were only seen in the S*pns2^tm1a^* and S*pns2^tm1b^* homozygous mutants. We did not see anterior eye defects in any of the 5 conditional alleles, consistent with normal anterior eye development in another conditional *Spns2;Tie2-Cre* mutant mouse [1]. The anterior eye phenotype appears to be due to a developmental abnormality resulting in defective eyelid formation and subsequent corneal opacity and vascularisation. *Spns2* also plays a role in retinal blood vessels. Our results showed that global *Spns2* knockout resulted in a mild phenotype of the retinal vasculature (thin and irregular veins) with decreased pericyte coverage in the central retina which may be related to the widely known role of S1P signalling in angiogenesis [76], [77]. The milder vascular phenotype in the retina than in the cochlea may be due to differences in the requirement for *Spns2* in these tissues. We also found focal retinal degeneration in these mutant eyes suggesting a role for *Spns2* in the photoreceptor and/or retinal pigment epithelium. Taken together, these findings suggest that *SPNS2* is not only a candidate gene for involvement in deafness, but also for deaf-blind syndromes.

In summary, we report here that *Spns2*-deficient mice displayed rapidly progressive hearing impairment associated with a rapid decline in the EP between P14 and P21. The mechanism by which Spns2 deficiency leads to decreased EP merits further investigation, but it most likely involves local S1P signalling. Following the early drop in the EP, later changes include reduced expression of key proteins involved in cochlear homeostasis and ultimately sensory hair cell loss. Our findings suggest that *Spns2* is a promising candidate gene for human deafness. Furthermore, *Spns2*-deficient mice may serve as a model to learn more about the role of S1P signalling in auditory function and the mechanism underlying at least one form of strial hearing loss.

## Materials and Methods

### Production and genotyping of *Spns2*-deficient and conditional knockout mice

Mouse studies were carried out in accordance with UK Home Office regulations and the UK Animals (Scientific Procedures) Act of 1986 (ASPA) under a UK Home Office licence, and the study was approved by the Wellcome Trust Sanger Institute's Ethical Review Committee. Mice were culled using methods approved under this licence to minimize any possibility of suffering. The mice were maintained in individually-ventilated cages at a standard temperature and humidity and in specific pathogen-free conditions. Either sex was used for this study.

The *Spns2* mutant allele we used carries a promoter-driven cassette designed to interrupt normal gene transcription but flanked by Frt sites to enable its removal and conversion to a conditional allele with a critical exon surrounded by LoxP sites (a knockout-first design; [21], [78]). The allele is designated *Spns2^tm1a(KOMP)Wtsi^*, abbreviated to *Spns2^tm1a^* in this study. A schematic of the knockout-first design of *Spns2^tm1a^* allele is shown in Fig. 1A. The mutant mice were generated by blastocyst injection of the targeted ES cell using standard techniques [20], [21] and germ line transmission of *Spns2^tm1a^* was confirmed by a series of genotyping PCR analyses [79].

The *Spns2^tm1a^* colony was maintained on a mixed genetic C57BL/6Brd*^Tyrc-Brd^*;C57BL/6Dnk;C57BL/6N background. *Spns2^tm1a/tm1a^* mice were crossed to *Hprt^Tg(CMV-Cre)Brd/Wtsi^* transgenic mice (on a C57BL/6NTac background) with systemic expression of Cre recombinase to remove the cassette and produce mice carrying the *Spns2^tm1b^* allele (Fig. 1A). Mice showing the correct excision were mated to wildtype C57BL/6N mice and offspring carrying the *Spns2^tm1b^* allele were mated to breed out the *Cre* allele and expand the colony. The *Spns2^tm1c^* allele was produced by crossing *Spns2^tm1a/tm1a^* mice to *Gt(ROSA)26Sor^tm1(FLP1)/Wtsi^* mice expressing Flp recombinase ubiquitously in which the promoter-driven cassette was excised and exon 3 was retained flanked by LoxP sites (Fig. 1A). All the genotyping PCR primers and product sizes are shown in Table 1. Lack of the *rd8* mutant allele was confirmed by conventional sequencing of the *Crb1* gene [33].

**Table 1:** Primers for genotyping.

Genotyping PCR	Primer Forward (5′ to 3′ order)	Primer Reverse (5′ to 3′ order)	Product Size (base pairs, bp)
*Spns2 wildtype*	CAAAACAATATGGGCTGGGG	GATGAAGGCAGGACTCAGGG	363
*Spns2^tm1c^*	CAAAACAATATGGGCTGGGG	GATGAAGGCAGGACTCAGGG	approx. 470
*Spns2^tm1a^*	CAAAACAATATGGGCTGGGG	TCGTGGTATCGTTATGCGCC	187
*Spns2^tm1d^*	CAAAACAATATGGGCTGGGG	TCGTGGTATCGTTATGCGCC	187
*LacZ*	ATCACGACGCGCTGTATC	ACATCGGGCAAATAATATCG	Wildtype:absent; Mutant:approx. 180
*Neo*	CAAGATGGATTGCACGCAGGTTCTC	GACGAGATCCTCGCCGTCGGGCATGCGCGCC	Wildtype:absent; Mutant:approx. 700
*Sox10-Cre*	GCGGTCTGGCAGTAAAAACTATC	GTGAAACAGCATTGCTGTCACTT	Wildtype:absent; Mutant:approx. 100
*Lyve1 Cre wildtype*	TGCCACCTGAAGTCTCTCCT	TGAGCCACAGAAGGGTTAGG	425
*Lyve1 Cre mutant*	GAGGATGGGGACTGAAACTG	TGAGCCACAGAAGGGTTAGG	210
*Epor Cre wildtype*	CAGGAATTCAAGCTCAACCTCA	GGCAGCCTGGGCACCTTCAC	431
*Epor Cre mutant*	CAGGAATTCAAGCTCAACCTCA	GTGTGGCTGCCCCTTCTGCCA	679
*Tie1 Cre*	GATGCCGGTGAACGTGCAAAACAGGCTC	CGCCGTAAATCAATCGATGAGTTGCTTC	Wildtype:absent; Mutant: approx. 400
*Pf4 Cre wildtype*	CAAATGTTGCTTGTCTGGTG	GTCAGTCGAGTGCACAGTTT	200
*Pf4 Cre mutant*	CCCATACAGCACACCTTTTG	TGCACAGTCAGCAGGTT	450

*Spns2^tm1c/tm1c^* mice were mated to *Sox10-Cre* mice (Tg(Sox10-cre)1Wdr) [31] to delete the floxed exon 3 and to generate a frameshift mutation of *Spns2* in the inner ear and craniofacial neural crest-derived tissues [31]. Genotyping was carried out using genomic DNA extracted from pinna tissue, which was mosaic under this conditional knockout strategy, and the conditional *Spns2^tm1d^* allele was confirmed by co-presence of *Spns2^tm1c^* and *Sox10-Cre* allele PCR bands. Since S1P is released from different blood cells and endothelial cells, we used mice expressing Tie1-Cre [80], Pf4-Cre [81], Lyve1-Cre [82] and EpoR-Cre [83] to inactivate the Spns2 gene in blood vessel endothelial cells, platelets, lymphatic endothelial cells and red blood cells respectively by crossing with *Spns2^tm1c/tm1c^* mice to produce the conditional knockouts.

### Real-time PCR

The organ of Corti, lateral wall (stria vascularis and spiral ligament), eyes and livers of postnatal day (P)4 homozygous, heterozygous and wildtype littermate mice were dissected in RNAlater (n = 3 for each genotype). Total RNA was isolated with QIAshredder columns (QIAgen, cat. no. 79654) and the RNAeasy mini kit (QIAgen, cat. no. 74104). RNA was normalized to the same concentration for cDNA synthesis using oligo dT and SuperScrip II (Invitrogen). Real-time PCR was performed in triplicate for each sample using a CFX connect real time PCR machine (BIO-RAD). The *Spns2* probe was designed to cover the 3′ untranslated region (Applied Biosystem). Hypoxanthine-guanine phospharibosyltransferase (Hprt) was amplified simultaneously (Applied Biosystem, Mm01318747_g1) as an internal reference. The relative quantity of *Spns2* was calculated using the 2^−ΔΔCt^ method [84].

### Reporter gene visualisation

X-gal staining can be used to visualise the expression of *Spns2* due to the *LacZ* gene inserted in the cassette of *Spns2^tm1a^* allele (Fig. 1A), downstream of the *Spns2* promotor. Inner ears of P10 and P14 heterozygous and homozygous mice (at least three mice of each age group) were dissected out and fixed in 4% paraformaldehyde (PFA) for 45 minutes to 2 hrs. These were washed twice with PBS and decalcified in 10% EDTA until soft. After a PBS wash and immersing in 30% sucrose, inner ears were embedded in Agarose type VII (low gelling temperature, Sigma-Aldrich), then mounted using OCT compound ready for cryosectioning at 14 µm. Sections were treated with Solution A (2 mM MgCl_2_; 0.02% NP-40; 0.01% sodium deoxycholate; PBS) for 15 mins, then incubated with Solution B (Solution A plus 5 mM K_3_Fe(CN)_6_; 5 mM K_4_Fe(CN)_6_; 1 mg/ml X-Gal in DMSO) over night at 37°C. Sections were rinsed in water then counterstained in Fast Red to label nuclei, mounted and examined.

### Auditory Brainstem Response (ABR)

Mice were anaesthetised by ketamine hydrochloride (100 mg/Kg, Ketaset, Fort Dodge Animal Health) and xylazine hydrochloride (10 mg/Kg, Rompun, Bayer Animal Health) and subcutaneous needle electrodes were inserted on the vertex (active), and over the left (reference) and right (ground) bullae. A calibrated sound system was used to deliver free-field click (0.01 ms duration) and tone pip (various frequencies from 6–30 kHz of 5 ms duration, 1 ms rise/fall time) stimuli at a range of intensity levels in 5 dB steps. Averaged responses to 256 stimuli, presented at 42.2 per second, were analysed and thresholds established as the lowest sound intensity giving a visually-detectable ABR response [85]. For P14 and P21 mice, in order to achieve higher sound pressure levels, sound was delivered to the external auditory meatus via a parabolic cone loud speaker attachment for click (0.01 ms duration) and tone pip stimuli (frequencies from 3–42 kHz of 5 ms duration in 3 dB SPL steps). Separate cohorts of P14 and P21 *Spns2^tm1a^* mice were used at the standard maximum intensity of 95 dB SPL with free field delivery as shown in Fig. 11 and at the higher sound intensities delivered near field, directly to the external auditory meatus in Fig. 1. The median ABR thresholds recorded in homozygous mutants at P14 and P21 shown in Fig. 1 were compared using the Kruskall-Wallis One-Way Analysis of Variance on Ranks, as thresholds did not show a normal distribution.

### Scanning electron microscopy and gross morphology of ears

The temporal bones were isolated. The inner ears were dissected out and fixed by 2.5% glutaraldehyde in 0.1M sodium cacodylate buffer with 3 mM calcium chloride at room temperature for 3 hours. Cochleae were finely dissected in PBS. This was followed by further processing using an osmium-thiocarbohydrazide-osmium (OTOTO) method [86]. The samples were dehydrated in increasing concentrations of ethanol, critical-point dried (Bal-Tec CPD030), mounted and examined under a HITACHI S-4800 scanning electron microscope. At least 3 wildtype, heterozygous and homozygous mice were examined for each age group (P4, P21, P28 and P56). Middle ears were dissected and examined. Inner ears were cleared by a standard glycerol clearing technique and examined for gross structural defects (control, n = 5; homozygotes, n = 5; aged P30–34).

### Semi-thin sections and transmission electron microscopy

Inner ears (wildtype, n = 2; heterozygotes, n = 2; homozygotes, n = 4, at P28) were dissected out and gently perfused with 2.5% glutaraldehyde, 1% paraformaldehyde in 0.1M sodium phosphate buffer with 0.8 mM calcium chloride through the round and oval windows and a small hole in the apex then fixed at room temperature for 2 hours. Secondary fixation was in 1% osmium tetroxide in sodium phosphate buffer for 1 hour. After 5 washes in 0.1 M sodium phosphate buffer, inner ears were decalcified in 0.1M EDTA at 4°C until soft. Then the samples were dehydrated through an ethanol series, staining in 2% uranyl acetate at the 30% ethanol stage, embedded in Epon resin mixed 1∶1 and then 3∶1 in propylene oxide for 30 min and infiltrated overnight under vacuum in neat resin. The samples were embedded at 60°C for 24–48 hours. 1 µm sections were cut through the modiolar plane and stained with toluidine blue for light microscope observation. 60 nm sections were cut on a Leica EM UC6 ultramicrotome, stained in 2% uranyl acetate and aqueous lead citrate and imaged on an FEI Spirit Biotwin 120 kV transmission electron microscope using a Tietz F4.15 CCD.

### Endocochlear potential measurement

Mice were anaesthetized with 0.01 ml/g body weight of 20% urethane, a tracheal cannula was inserted and the bulla was opened to reveal the cochlea while the body temperature was kept at 37°C by a feedback-controlled heating pad. A small hole was made in the bony wall of the cochlea over the basal turn of scala media, and a micropipette electrode filled with 150 mM potassium chloride was advanced through the hole and through the lateral wall into the scala media. The potential difference between the scala media and a reference silver/silver chloride pellet under the dorsal skin was recorded [24].

### Surface preparation of lateral wall and visualization of capillaries

The inner ears were rapidly dissected out and fixed in 4% paraformaldehyde at room temperature for 2 hours. The lateral walls were dissected out in PBS for surface preparation. Filamentous actin was visualized by rhodamine phalloidin (1∶200, Molecular Probe) at room temperature for 2 hours. Strial capillaries were visualized by Isolectin B4 (Vector Laboratories, 1∶50) at 4°C, overnight in PBS with 10% sheep serum). Samples were mounted with Vectashield Mounting medium (Vector, Cat. No: H-1000) and imaged by confocal microscopy (Carl Zeiss, LSM 510 META). The numbers of capillary branch points per field (220×220-µm fields) in the middle turn (40–70% of the distance along the cochlear duct from the base) of the stria vascularis (control, n = 4; homozygotes, n = 5, at P14. control, n = 3; homozygotes, n = 5, at P28) was quantified using image J. Data were presented as a density in a 100×100 µm field and statistics analysis was conducted using Mann-Whitney Rank Sum Test, SigmaPlot v12.0. Surface preparations were also used for analysis of Kcnq1 expression, using overnight incubation at 4°C with goat anti-Kcnq1 polyclonal antibody (Santa Cruz, 1∶200) followed by washing with PBS and incubation with donkey anti-goat secondary antibody (Invitrogen, 1∶500) prior to analysis by confocal microscopy. At least three homozygotes and three controls were used at P14, P28 and 6 months for phalloidin labeling to show marginal cell boundaries, and P14 and 5–6 weeks for Kcnq1 expression. The density of marginal cells was measured in the phalloidin-labelled whole mount preparations by counting the number of cells defined by their labeled boundaries in two areas each 100×100 µm from the middle turn (40–70%) of each cochlea (n = 4 homozygous mutants; 4 littermate controls).

### Immunofluorescence labeling

The cochleae were dissected out and fixed in 4% PFA at room temperature for 2 hours. Cryosections were obtained as described above for X-gal staining. We used the following antibodies: rabbit anti-Kcnj10 polyclonal (Alomone labs, 1∶300), rabbit anti-Gjb2 polyclonal (from WH Evans, 1∶300), rabbit anti-Gjb6 polyclonal (Zymed, 1∶400), mouse anti- Na^+^, K^+^-ATPase (α1 subunit) monoclonal (Sigma, 1∶300), mouse anti- NKCC1 monoclonal (C. Lytle, Developmental Studies Hybridoma Bank, University of Iowa, 1∶300) and rabbit anti-ZO-1 polyclonal (Zymed, 1∶300). Sections were blocked by incubation with 10% sheep serum (with 0.1% TritonX-100 in PBS) for 40 mins. Sections were incubated with appropriate primary antibodies overnight at 4°C, washed with PBS and incubated with corresponding secondary antibodies at room temperature for 2 hours (donkey anti-rabbit, donkey anti-goat, Invitrogen, 1∶500). After washing with PBS, slides were imaged by confocal microscopy. Three mice of each genotype (*Spns2^tm1a/tm1a^* and *Spns2^+/+^*) were used for each antibody at P14 and 5–6 weeks old.

### Stria vascularis tight junction permeability assessment

The inner ears (wildtype, n = 1; heterozygote, n = 3; homozygote, n = 4 at 6 weeks old) were dissected and round and oval windows were opened in PBS containing 1 mM calcium chloride. A hole was made in the basal turn leading to the scala media. The membranous labyrinth was perfused for 5 minutes with 400 µl Sulfo-NHS-LC-Biotin (Thermo Scientific, 10 mg/ml, in PBS with 1 mM calcium chloride) through the round and oval windows and the hole exposing the endolymphatic compartment. Following a PBS wash, the inner ears were fixed in 4% paraformaldehyde at room temperature for 2 hours, and processed for cryosectioning as described above. The biotin tracer was detected by fluorescein isothiocyanate (FITC)-conjugated streptavidin (Thermo Scientific, 1∶100) incubating at room temperature for 30 min. Samples perfused with PBS alone were used as negative controls.

### Strial capillary permeability assessment

Mice (wildtype, n = 2; heterozygote, n = 2; homozygote, n = 2; at 5–6 weeks old) were warmed in a 39°C incubator for 5 minutes and then held in a mouse restrainer so that the tail was accessible and the tail vein visible. A 50 µl aliquot of 5% (w/v) FITC-conjugated bovine serum albumin (BSA-FITC; Sigma cat. no. A9771; size 66.4 kDa), made up in sterile PBS, was injected into the tail vein. The mice were left at room temperature for 45–60 minutes to allow the BSA-FITC to permeate all capillaries and to allow for any vascular extravasation. The mice were sacrificed by CO_2_ inhalation and the auditory bullae dissected out. Whole cochleae were exposed and fixed by removing a small piece of bone at the apex and gently perfusing 4% PFA through the round and oval windows. Cochleae were then immersed in fixative and left on a rotator for 1.5 hours at room temperature. Whole-mounts of stria vascularis were dissected from fixed cochleae, covered with Vectashield Mounting Media (Vector, Cat. No: H-1000) in glass bottom culture dishes (MatTek Corp.) and imaged using confocal laser-scanning microscopy (Carl Zeiss, LSM 510 META).

### Ocular assessment

Mice underwent ophthalmic screening at 15 weeks of age. They were assessed for gross morphological changes to the eye using a slit lamp (Zeiss SL130) and ophthalmoscope (Heine Omega 500). The eye was examined both undilated and dilated (topical tropicamide). Images using the slit lamp were collected using a Leica DFC420 camera. The mice were culled under terminal anaesthesia followed by cervical dislocation and both eyes from 3 male homozygous mutants and 3 wildtype mice were removed and fixed. Pupil-optic nerve sections were processed, stained with hematoxylin and eosin, and standard images were captured under light microscopy for review [87].

For whole mount retinal analysis, heterozygotes and homozygotes were used (n = 3 for each genotype at P10, n = 2 at 8 weeks old) and the eyes removed and fixed in 4% PFA. Retinae were prepared and stained as described [88] using flourescein-conjugated Griffonia simplicifolia Isolectin B4 (Vector Laboratories, UK) to label blood vessels, mouse anti-proteoglycan NG2 (Millipore UK Ltd., UK) to label pericytes, and donkey anti-rabbit secondary Alexa-594 (Molecular Probes, Life Technologies, UK). The homozygote and control mouse retinae were stained in the same well to control for changes in staining efficiency, distinguishing the retinae by different numbers of radial incisions. All tissues were mounted in Vectashield (Vector Laboratories Ltd., Peterborough, UK), imaged by confocal microscopy (Nikon A1R; Nikon Instruments, Inc., Melville, NY), and maximum intensity projections of z-stacks were created using NisElements AR Version 4.0 software (Nikon UK, Kingston Upon Thames, UK). The MetaMorph Angiogenesis Tube Formation application (Molecular Devices, Berkshire, UK) was used for quantification. Confocal images were used to determine the total area covered by vessels and by pericytes to calculate the percentage pericyte coverage of vessels and the total number of capillary branchpoints per unit area. We imaged three areas of each retina: three different regions of the central retina each encompassing an artery and vein, and two images each of peripheral arteries and peripheral veins, a total of five images/retina. Threshold values were kept the same for analysis of samples of the same stage.

## Supporting Information

Figure S1 *Spns2* expression in the vestibular system, normal gross structure of inner ears and normal organ of Corti at P4. ***A,B***: X-gal staining showed expression of *Spns2* in the vestibular system at P10. Labelling (blue) was detected in the cristae (A, arrow) and maculae (utricular macula shown here) (B, arrow). Scale bar: 20 µm. ***C,D***: Cleared inner ears showed no apparent differences in gross structure between *Spns2* homozygous mutants and controls at 4 weeks old. Scale bar: 1 mm. ***E,F***: Scanning electron microscopy showed no abnormalities of the surface of the organ of Corti at P4 in the *Spns2* homozygous mutants compared with littermate controls. Scale bar: 10 µm.
(TIF)

Figure S2 Whole-mount stria vascularis examination. ***A,B***: Confocal images focussed at the level of the basal cell boundaries, visualised by phalloidin staining (red). No obvious change was seen in *Spns2* homozygous mutants at 4 weeks old. Scale bar: 10 µm. ***C,D***: The stria vascularis showed dilated and tortuous capillaries with increased branch points in all five *Spns2* homozygous mutants studied at 4 weeks old. Strial capillaries were visualised by isolectin B4 (green). Scale bar: 50 µm. ***E,F***: Strial hyperpigmentation was pronounced in older mutants. A seven month old *Spns2* homozygous mutant (***F***) had obvious accumulation of pigment in the stria vascularis. Scale bar: 20 µm.
(TIF)

Figure S3 Normal expression of Na^+^/K^+^-ATPase, NKCC1 and ZO-1 in lateral wall and Kcnj10 in spiral ganglion at 5–6 weeks. Na^+^/K^+^-ATPase (red) labelling in stria vascularis and type II fibrocytes in *Spns2* homozygous mutants (***A***) was comparable with that of controls (***B***). Notice absence of Gjb2 labelling in the type II fibrocytes in the mutants. NKCC1 (red) labelling in the *Spns2* homozygous mutants was located in stria vascularis and type II fibrocytes and appeared similar in the controls (***C***, ***D***). ZO-1 (green) labelling was present in the basal cells of the stria in both *Spns2* homozygous mutants and controls (***E,F***). ***G,H***: Acetylated α-tubulin (red) labelled spiral ganglion neurons and Kcnj10 (green) labelled satellite cells. Kcnj10 expression in *Spns2* homozygous mutants was present and comparable with controls, suggesting that the reduced Kcnj10 labelling observed in the stria (see Fig. 8) was tissue-specific. Scale bar: 20 µm in A–H.
(TIF)

Figure S4 Pericyte coverage of retinal blood vessels and open eyelids at birth. ***A***: Analysis of the percentage of pericyte coverage of the retinal blood vessels revealed a significantly reduced coverage in the mutants in the central retina (t-test for central vessels; p = 0.015), but no significant difference in coverage of the peripheral vessels (arterial and venous) (Mann-Whitney Rank Sum Test for peripheral arterial vessels, p = 0.151; t-test for peripheral venous vessels, p = 0.284) between the *Spns2* mutant homozygotes and heterozygous controls at P10. ***B***: *Spns2^tm1a^* homozygous mutants displayed open eyelids at birth.
(TIF)
