# Supplementary figures and images for: Spinster Homolog 2 (Spns2) Deficiency Causes Early Onset Progressive Hearing Loss
Source: PLoS Genet. 2014 Oct 30;10(10):e1004688. doi: 10.1371/journal.pgen.1004688 (PMC4214598; doi:10.1371/journal.pgen.1004688)

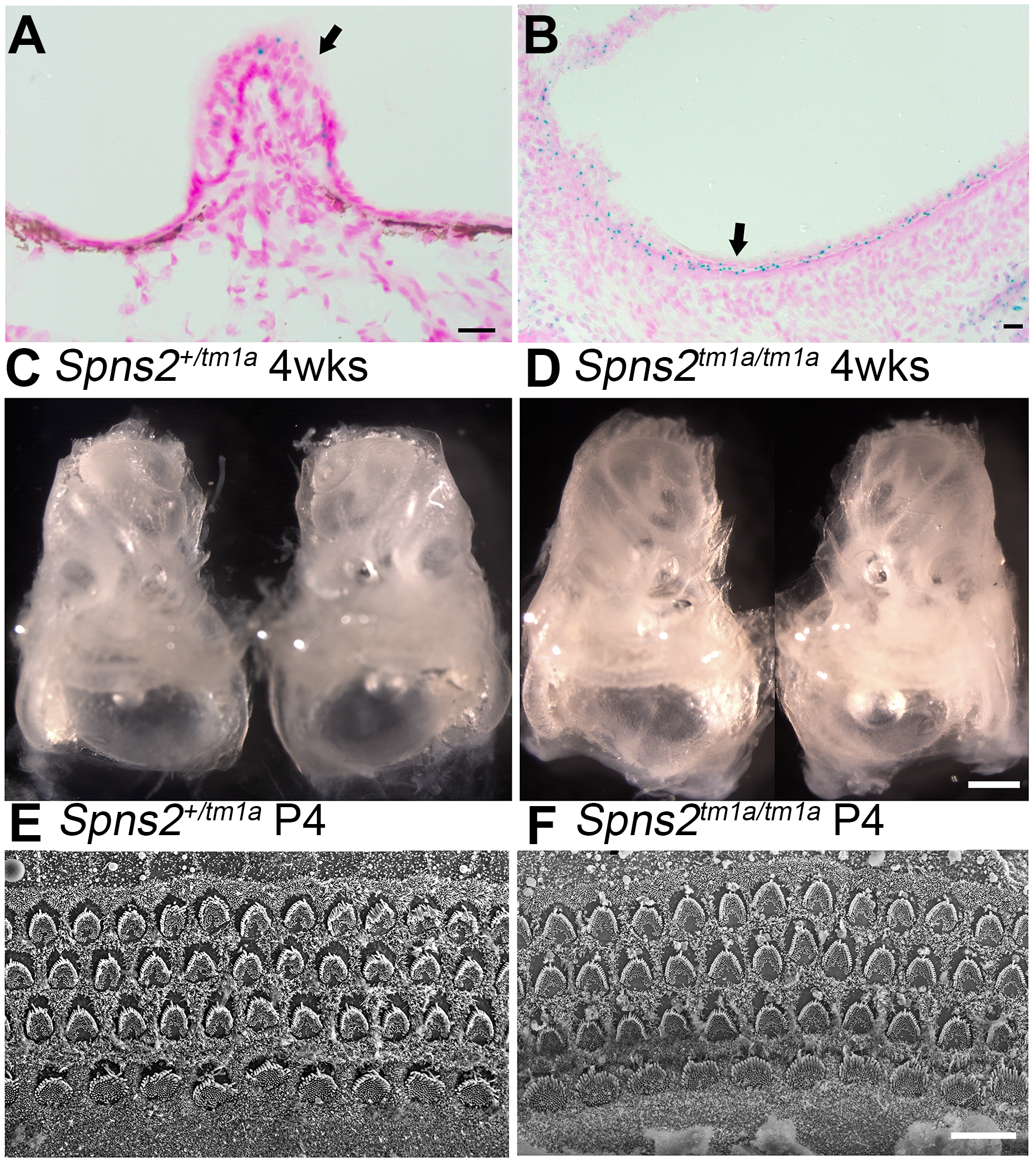

Supplement: Figure S1 — Spns2 expression in the vestibular system, normal gross structure of inner ears and normal organ of Corti at P4. A,B: X-gal staining showed expression of Spns2 in the vestibular system at P10. Labelling (blue) was detected in the cristae (A, arrow) and maculae (utricular macula shown here) (B, arrow). Scale bar: 20 µm. C,D: Cleared inner ears showed no apparent differences in gross structure between Spns2 homozygous mutants and controls at 4 weeks old. Scale bar: 1 mm. E,F: Scanning electron microscopy showed no abnormalities of the surface of the organ of Corti at P4 in the Spns2 homozygous mutants compared with littermate controls. Scale bar: 10 µm. (TIF) [file pgen.1004688.s001.tif]

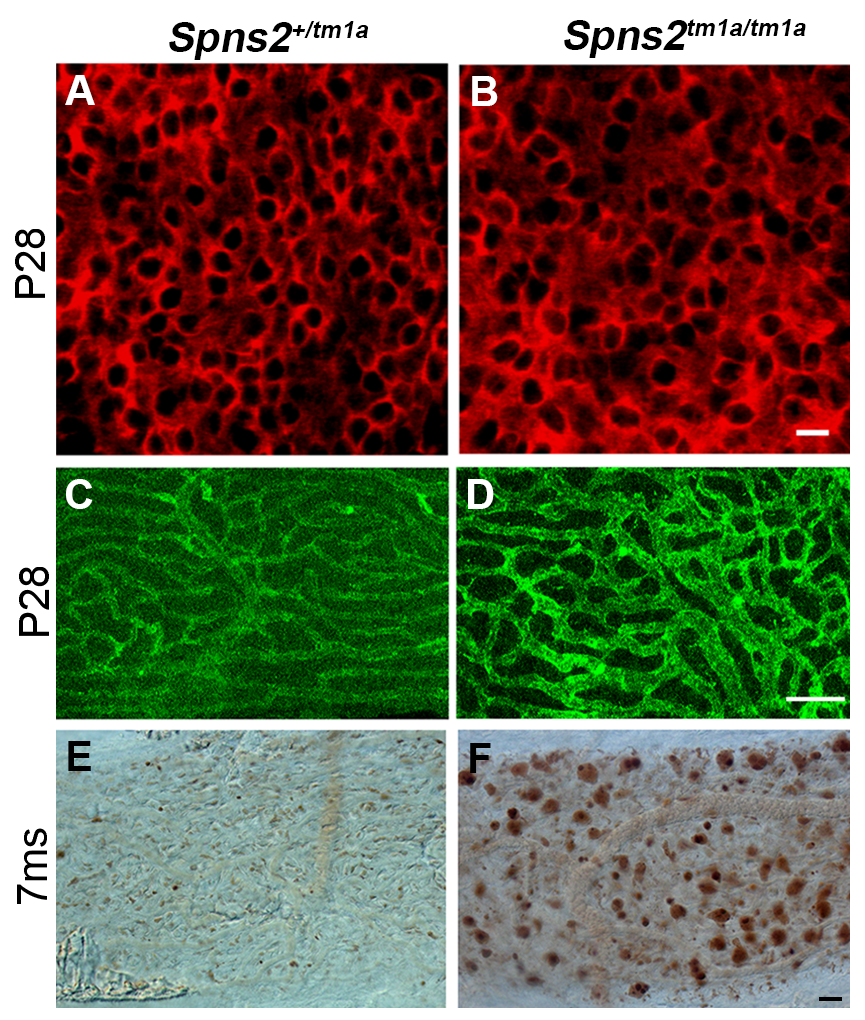

Supplement: Figure S2 — Whole-mount stria vascularis examination. A,B: Confocal images focussed at the level of the basal cell boundaries, visualised by phalloidin staining (red). No obvious change was seen in Spns2 homozygous mutants at 4 weeks old. Scale bar: 10 µm. C,D: The stria vascularis showed dilated and tortuous capillaries with increased branch points in all five Spns2 homozygous mutants studied at 4 weeks old. Strial capillaries were visualised by isolectin B4 (green). Scale bar: 50 µm. E,F: Strial hyperpigmentation was pronounced in older mutants. A seven month old Spns2 homozygous mutant (F) had obvious accumulation of pigment in the stria vascularis. Scale bar: 20 µm. (TIF) [file pgen.1004688.s002.tif]

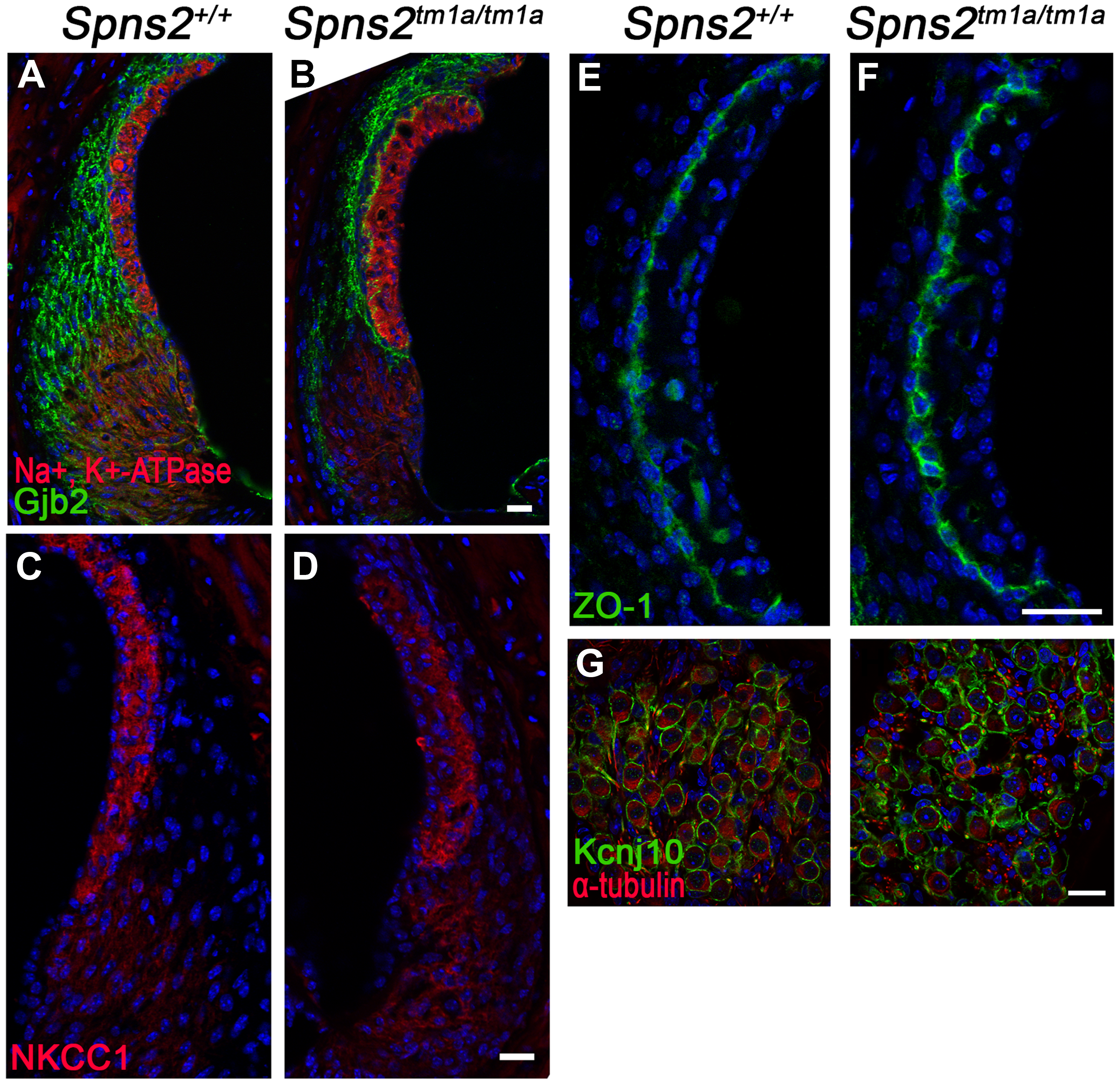

Supplement: Figure S3 — Normal expression of Na+/K+-ATPase, NKCC1 and ZO-1 in lateral wall and Kcnj10 in spiral ganglion at 5–6 weeks. Na+/K+-ATPase (red) labelling in stria vascularis and type II fibrocytes in Spns2 homozygous mutants (A) was comparable with that of controls (B). Notice absence of Gjb2 labelling in the type II fibrocytes in the mutants. NKCC1 (red) labelling in the Spns2 homozygous mutants was located in stria vascularis and type II fibrocytes and appeared similar in the controls (C, D). ZO-1 (green) labelling was present in the basal cells of the stria in both Spns2 homozygous mutants and controls (E,F). G,H: Acetylated α-tubulin (red) labelled spiral ganglion neurons and Kcnj10 (green) labelled satellite cells. Kcnj10 expression in Spns2 homozygous mutants was present and comparable with controls, suggesting that the reduced Kcnj10 labelling observed in the stria (see Fig. 8) was tissue-specific. Scale bar: 20 µm in A–H. (TIF) [file pgen.1004688.s003.tif]

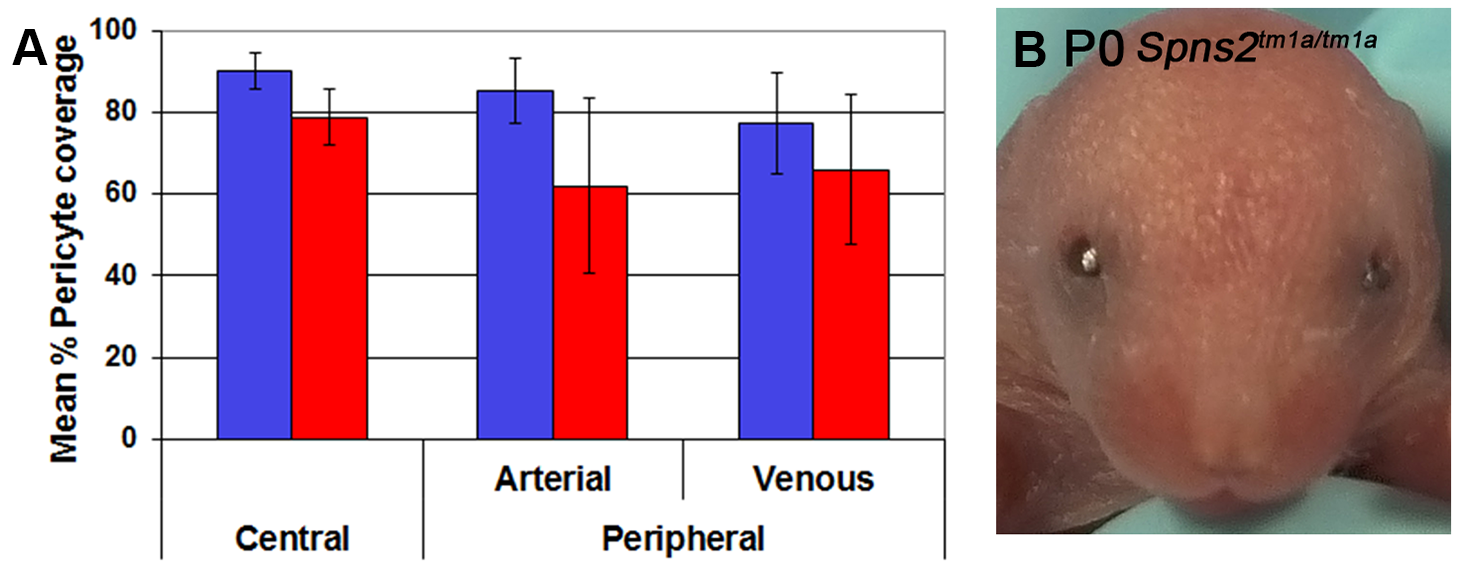

Supplement: Figure S4 — Pericyte coverage of retinal blood vessels and open eyelids at birth. A: Analysis of the percentage of pericyte coverage of the retinal blood vessels revealed a significantly reduced coverage in the mutants in the central retina (t-test for central vessels; p = 0.015), but no significant difference in coverage of the peripheral vessels (arterial and venous) (Mann-Whitney Rank Sum Test for peripheral arterial vessels, p = 0.151; t-test for peripheral venous vessels, p = 0.284) between the Spns2 mutant homozygotes and heterozygous controls at P10. B: Spns2tm1a homozygous mutants displayed open eyelids at birth. (TIF) [file pgen.1004688.s004.tif]
